# Innovation concentration in knowledge network

**DOI:** 10.1371/journal.pone.0266530

**Published:** 2022-04-06

**Authors:** Jifeng Zheng

**Affiliations:** School of Economics, Shanghai University of Finance and Economics, Shanghai, China; Universiti Malaysia Sabah, MALAYSIA

## Abstract

This paper studies the increase in innovation concentration levels of U.S. industries over the last two decades. I present a model of imperfectly competitive patent market with heterogeneous firms that generate endogenous variable markups. Theoretically and empirically, the price of a firm’s newly invented knowledge, the profit and survival rate of the innovating firm depends on the market share of this firm’s knowledge stock and the position of industry in the knowledge network. In the process of innovating, firm pays a random fixed cost in each period which together with market share largely determines its decision on whether to innovate into different sectors. I find that firms with larger market share not only invest more in R&D but also enter into more sectors. Since innovating firms could create blueprints for new varieties and manufacture the products that have been invented, I bridge the gap between the product and the R&D markets to document the similar concentration trends between them. I prove that large firms in the product market would charge higher price on their product so that they can charge higher profit which is a part of the value of their knowledge. Lastly, the increasing trend of concentration could decrease consumers’ welfare in the industries with high initial concentration levels.

## Introduction

Product markets have undergone a structural change that had potentially transformed the nature of competition over the recent decades [1–3]. This paper shows that the phenomenon that the product market has become more concentrated across U.S. industries also occurs in R&D and patent markets since the beginning of the 21st century. The majority of theoretical and empirical works focus on the impact of industry concentration or industry competition on R&D [4–6]. On the contrary, this paper focus on the impact of R&D on industry concentration. I Show that R&D itself, under certain conditions, will increase industry concentration.

Empirical work on product market [1] suggests that the deregulation of antitrust policy enforcement could have direct implications on the product market competition. Different from their work which mainly focuses on the impact of deregulation on product market competition through M&A, I show that the rise of market power in R&D and patent markets could happen through the channel of R&D process itself. And further promote the increase in product market concentration.

The imperfectly competitive patent market plays a key role in explaining this self-fulfilment mechanism. In the context of an imperfectly competitive patent market, the price of firm’s newly invented knowledge, the profit and survival rate of the innovating firm depends on the market share of this firm’s knowledge stock and the position of industry in the knowledge network. Product and R&D markets are not completely separated, on the contrary, they are mutually complementary. I also show that this is true empirically and theoretically.

This paper pursues two goals. First, I document several stylized facts that motivate the research. I use R&D/patenting data to document the following facts: (1) the number of innovating firms start to decline from 1999; (2) the industry concentration, HHI as our proxy index, starts to increase across most of the industries since around 1999; (3) firms allocate more R&D resources into existing central industries, together with expanding business into new sectors; (4) product and R&D markets have similar concentration trends. The second objective is to develop a general equilibrium model of multi-sector firm innovation to explain these facts and to draw aggregate implications.

Traditionally, innovations are viewed as taking place in isolation. In the real world, however, innovation almost never happens in a single firm. Innovating firms adopt knowledge from different firms and sectors to create new ones in a knowledge network. Contrary to many theoretical works on endogenous growth which treats technologies as equally influential, this paper pays attention to various patent adoptions and different knowledge pricing strategies of heterogeneous firms in an imperfectly competition patent market. The market power of these firms, or essentially, the market structure, determines how firms allocate resources and conduct research. Imperfectly competition appears to describe many markets more accurately than perfectly competition: a variety of market structures exist. But how important is the imperfectly competition, in particular, the oligopolistic competition market structure for understanding firms’ distinct pricing strategies and different decisions on whether to innovate or which sectors to innovate in? This is the main question analyzed in this paper.

Intuitively, larger firms with a large share of blueprints in a sector have more market power on pricing, and thus they have more profit on innovation and production. Moreover, they will survive longer relative to smaller firms. However, the typical model is restrictive regarding the nature of competition within a sector: firms are large enough to have market power, but they behave as if prices were given. That is to say, firms do not internalize their market power when they make decisions. In this paper, I explore the oligopolistic market structure of sectors in which firms do take into account the effect of their decisions on patent price and quantity through the knowledge network.

The firm-level behavior of patenting with each other in the knowledge network is a key characteristic in this paper. Empirical evidence shows that most patent citations and investments in R&D take place between different knowledge sectors [7]. More than 50% of patenting firms innovate in more than one technological area.

More specifically, to explain the fact that firms choose specific industry to innovate, I present my model in a knowledge network framework. A knowledge network is a directed network of patent citations, which point from the patent citing sectors to cited sectors. If we equate patents with knowledge and innovation, we can use firm-level patent citation relationship to interpret innovating firm’s R&D strategies.

In many literatures on innovation and growth, firms buy patent application rights from other firms to create new knowledge in a perfect competition market. Most of these papers are based on the assumption of monopolistic competition and CES preferences as in [8], which imply that individual firms charge constant markups over their marginal costs. While being extremely convenient from the analytical point of view, constant markups are at odds with the empirical evidence. Moreover, constant markup models ignore the significantly different pricing power of heterogeneous innovating firms in the same sector. Different firms are endowed with distinct amount of knowledge stock. Firms with larger share of knowledge stock possess dominant positions in the sector will charge higher prices than smaller ones.

In this paper, I consider an oligopolistic competitive patent market to introduce heterogeneity in which firms would bid a firm specific knowledge price depending on the amount of knowledge stock share and the set of sectors that firm innovates in. The model is based on variety-expanding rather than quality ladder setup: firms invent new blueprints by adapting prior knowledge in various sectors through R&D. Applicable technologies can not only increase the innovative productivity of R&D but also contribute to a range of innovations in many sectors. When adapting prior knowledge, firms can utilize their own private knowledge, public knowledge and obtained licenses to use the private knowledge of other firms in various sectors. The latter takes place in an imperfectly competitive licensing market. Specifically, should any firm decides not to innovate in sector *i* in one period, it can–and finds it optimal in equilibrium to–license the application rights of its prior knowledge of sector *j* to other innovating firms in sector *i* during that period, assuming perfect intellectual property rights protection. Within each sector, a finite number of heterogeneous firms are subject to oligopolistic competition and set variable markups à la [9]. The markup charged is increasing in the patent share of the firm: larger firms charge a higher markup.

A firm has to pay an idiosyncratic fixed cost when conducting research in any given sector at any period. If a firm does not pay this cost, then it ceases to develop new blueprints in that sector. This continuation cost can be interpreted as a license fee or the financial cost of maintaining a research lab or entry cost. The equilibrium licensing fees that clear the market thus reflect the “application value” of the source knowledge *j* in innovating in sector *i*. The existence of a knowledge market thus allows all knowledge to be utilized in equilibrium, either by its original inventor or by other firms that have acquired its application rights. Therefore, the equilibrium value associated with knowledge capital in sector *j* is not only determined by the profit it generates in its own sector as in conventional models, but also depends on its application value in all sectors. Higher application value together with higher market share attracts firms to invest in R&D in that sector. A firm would only conduct research in a sector if its knowledge is sufficiently applicable and market share is large enough to generate a larger expected value than the fixed costs. Since the fixed cost faced by each firm is stochastic and idiosyncratic, the decision on research is only determined by the expected value of innovation which is also an increasing function of firms’ knowledge stock share.

In any given sector, incumbent firms innovate and expand their sizes as they create new varieties. However, they will also pause or stop innovating after experiencing a sequence of adverse R&D shocks or high fixed costs. This generates endogenous sorting of firms. The larger firms’ expected probability of innovating is larger. And they would find it’s easier to expand, but smaller firms would find it’s difficult to survive when the cost of entry is rising. Nevertheless, once succeed, they are more likely to defend their status. As a result of increasing entry cost, the number of innovating firms start to decline (*Fact 1*).

In addition, potential innovators would enter if they face low fixed costs and have accumulated enough knowledge capital–either by creating its own knowledge or by acquiring external knowledge–in related sectors. Incumbent firms are more likely to innovate in oligopolistic competition environment. In monopolistic competition or perfectly competition environment, firms are too small to possess market power. Therefore, larger firms with larger share of blueprints in a sector have more market power on pricing and more profit of research. This caused polarization in R&D: firms with larger market share develop more blueprints while smaller firms are just the opposite or even exit (*Fact 2*).

Moreover, as firms grow and accumulate more private knowledge, they extend their tenacles to all accessible and promising sectors. Surpringly, innovating firms enter more new sectors when entry costs are increasing. As entry costs rising, various resources occupied by firms become relatively scarce. However, the firm could contribute its own profits to help other research projects that are facing difficulties or are opening up new market shares to overcome difficulties. I will show that monopoly profits can cover entry costs (*Fact 3*).

Since innovating firms could create blueprints for new varieties and manufacture the products that have been invented, I bridge the gap between the product market and the R&D market. As interpreted in the model, on the one hand, large firms in the R&D market are still large firms in the product market, and large firms in the product market would charge higher price on their product so that they can charge higher profit which is a part of the value of their knowledge on the other hand. This explains why both markets would have similar trends in industry concentration (*Fact 4*).

### Related literature

This paper’s findings are relevant and important to the existing research on the evolution of market competition. [1] documents that over the last 15 years the level of product market concentration in the U.S. has increased across most industries. They explain this phenomenon as consolidation of public firms into mega firms. I find similar phenomenon in the R&D and patent market. [10] document empirical patterns to assess that if globalization or technological changes advantage the most productive firms in each industry, product market concentration will rise as industries become increasingly dominated by superstar firms with high profits and a low share of labor in firm value-added and sales. Similar but different from [2, 10] prove that the rise in national industry concentration in the U.S. between 1977 and 2013 is only seen in three broad sectors and is driven by a new industrial revolution. They present a model to describe the availability of a new set of fixed-cost technologies that enable adopters to produce at lower marginal costs in all markets.

[11] study the entry and exit of firms across U.S. industries over the past 40 years and they conclude that lobbying and regulations have caused free entry to fail. [12] show that the rise in intangibles is driven by industry leaders and coincides with increase in their market share and hence, rising industry concentration. [3] show that the interplay of population and firm demographics can account for the decrease in startup rates, firm exit rates and the increase in average firm size in the US. My paper emphasizes that imperfectly competition in patent market is a possible new important channel to explain innovation industry concentration.

Recently, there are many theoretical and empirical literatures about models of monopolistic competition featuring variable markups. [13, 14] focus on additively separable preferences, while [15, 16] embed a non-separable quadratic utility into a quasi-linear framework. These models mentioned above have proven to be tractable in a static framework, as noted by [17], while extending them to a dynamic growth model is however impossible.

A second strand of the literature about endogenous variable markups is based on market structure. [9] use a nested constant elasticity substitution demand system that generates variable markups under imperfectly competition focusing on international pricing. [18] builds a tractable multi-sector heterogeneous-firm general equilibrium model featuring oligopolistic competition and input-output network. He mainly focuses on firm-level productivity shocks can lead to sector- and macroeconomic-level outcomes. Moreover, the structural importance of a firm is determined by the interaction of the sector-level competition intensity, the firm’s sector position in the I-O network and the firm size. Relative to these papers, I introduce imperfectly competition structure into knowledge market in which firms innovate by applying existing knowledge in related sectors.

Finally, this paper relates to a strand of literature about multi-product firms. I treat multi-product firms as firms that innovate multiple patents or innovate into different industries and sectors. [19] examines a new, “extensive margin” of firm adjustment, the reassignment of resources that takes place within surviving firms as they add and drop (i.e., “switch”) products. Empirical examination of firms’ product mix, however, reveals that firms are more likely to co-manufacture products within the same industry, or within “linked” industries. [20] presents a new model of multi-product firms and flexible manufacturing, in which they predict that selection effects operate at the product level, with firms encouraged to focus on their “core competence” and drop marginal high-cost varieties. [21] connect growth theories with findings from firm-level and sectoral-level studies of innovation. [22] study a model of innovation within multi-sector firms, with a focus on internal versus external innovation and its relation with firm size distribution.

The structure of our paper is as follows. I first document several stylized facts about innovation concentration. Next, I set up a model of consumption and production. Then I propose the framework of innovation in an incomplete competitive license market. The final section concludes this paper.

## Stylized facts

In this section, I first document several stylized facts that motivate my model using firm patenting and R&D investment data. My main sample about R&D expenditure consists of all firms in the Compustat database over the period of 1976–2018. I focus attention on firms incorporated in the U.S. that trade on major stock exchanges. Throughout the paper, I use NAICS classification to define a firm’s industry rather than SIC. Since NAICS codes are based on a consistent grouping among establishments that use the same or similar production process. Under the SIC system, some establishments are classified according to production process, but others are classified using different criteria. Using SIC codes, whenever possible, does not qualitatively affect any of the results. To analyze the R&D trend, I construct the Herfindahl-Hirschman concentration index (*HHI*). To construct the HHI, within every NAICS 2-digit industry-year, I sum up the squared ratios of firm sizes to the total industry sizes. Following the approach in [23], I also construct the HHI in the entire economy. I assign the industry-level HHI to each firm, essentially weighting each industry ratio by the number of public firms, and then aggregate across firms in every year.

My main data source about patent is NBER patent data project and USPTO from 1976 to 2006 which consists of patent citations and firm patenting (see [24] for details). I use this dataset to characterize firms’ innovation activities and their citations to trace the direction and intensity of knowledge flows and to construct indices of knowledge linkages among sectors. I summarize each firm’s patent stock in each disaggregated technological class (intensive margin of innovation) and the number of categories (extensive margin of innovation) for each year. Each patent corresponds to one of the 428 3-digit United States Patent Classification System (*USPCS*) technological classes and also one of the 37 2-digit technology subcatgories.

To obtain information of firm sizes (i.e. number of employees, sales), I use the NBER’s mapping between the Compustat data and the patent data between 1976 to 2006 and keep only patenting firms.

### Fact 1. Change in the number of innovating firms

**Fig 1:**
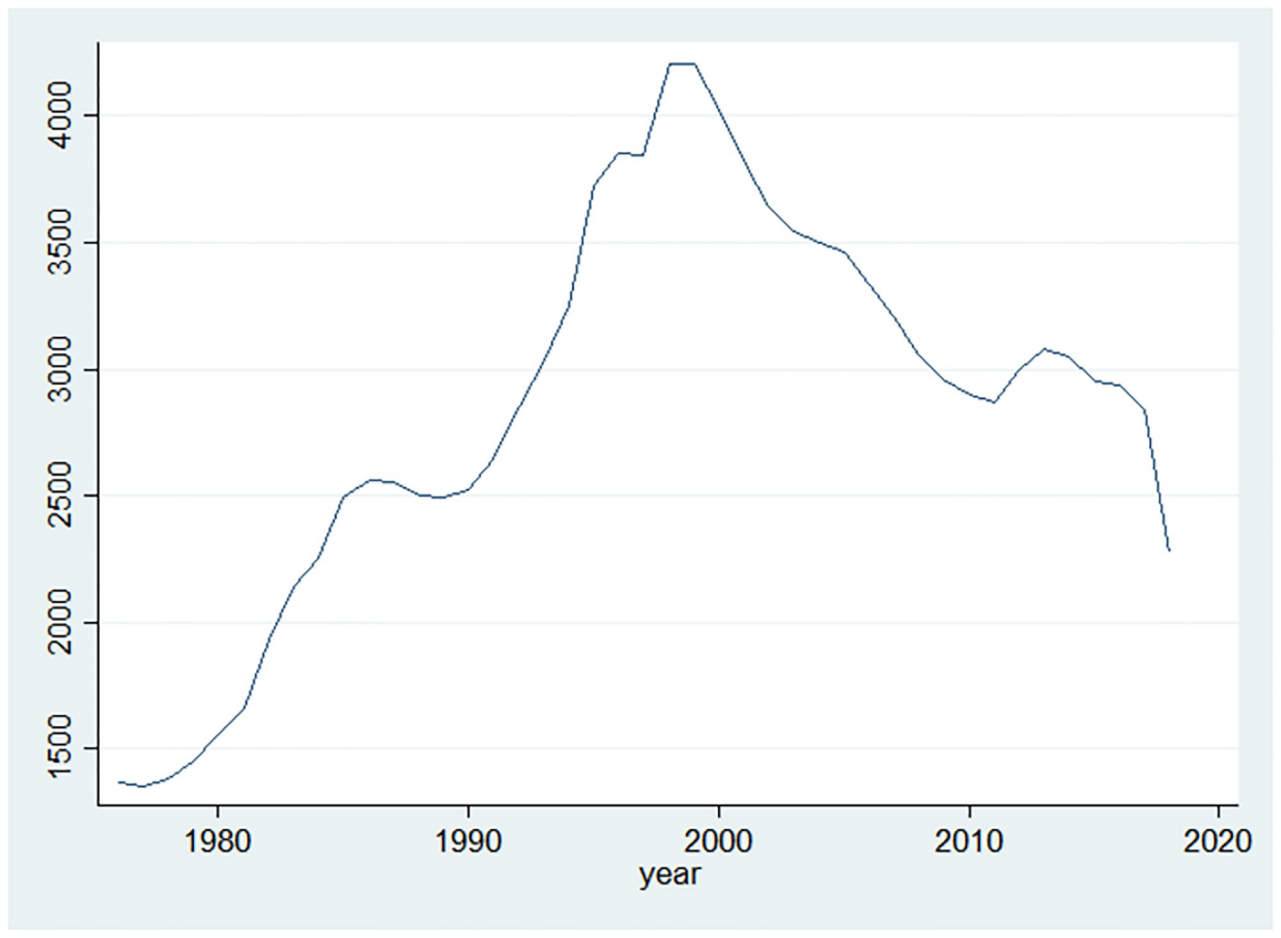
Number of firms.

Fig 1 shows that the number of innovating firms steadily increased during the first part of the sample (1976–1998). In the later period, however, the number of firms significantly declined from 4208 in 1998 to 2275 in 2018. This decline has been so substantial that the current number of firms in the economy is similar to the one in mid 1980s.

Table 1 shows the change in the number of innovating firms in the later period for each broad industry. I calculate the percentage change in the number of firms in each industry during the 1999–2017 period. I use 1999 as our starting period for the reason that 1998–2000 are the years in which the number of firms in our sample peaks. The decrease in the number of firms is prevalence in all of the industries. This table echoes [1]’s finding that the number of publicly traded firms that trade on major stock exchanges significantly declined over the similar period whose sample is based on the CRSP-Compustat dataset. Since over half of the firms are in the manufacturing industry, the percentage change in the manufacturing industry is similar with the all industries’ one.

**Table 1:** Percentage change in the number of firms by industries, 1999–2017.

Average of All Industries	-0.32	Real Estate Rental and Leasing	-0.7
Professional, Scientific Services	-0.69	Utilities	-0.67
Retail Trade	-0.65	Wholesale Trade	-0.6
Construction	-0.55	Information	-0.46
Agriculture, Forestry and Fishing	-0.46	Services	-0.42
Mining	-0.33	Finance and Insurance	-0.3
Manufacturing	-0.23	Transportation and Warehousing	-0.14

Note: The percentage change in the number of firms in each industry during the 1999–2017 period

### Fact 2. Increase in industry concentration

**Fig 2:**
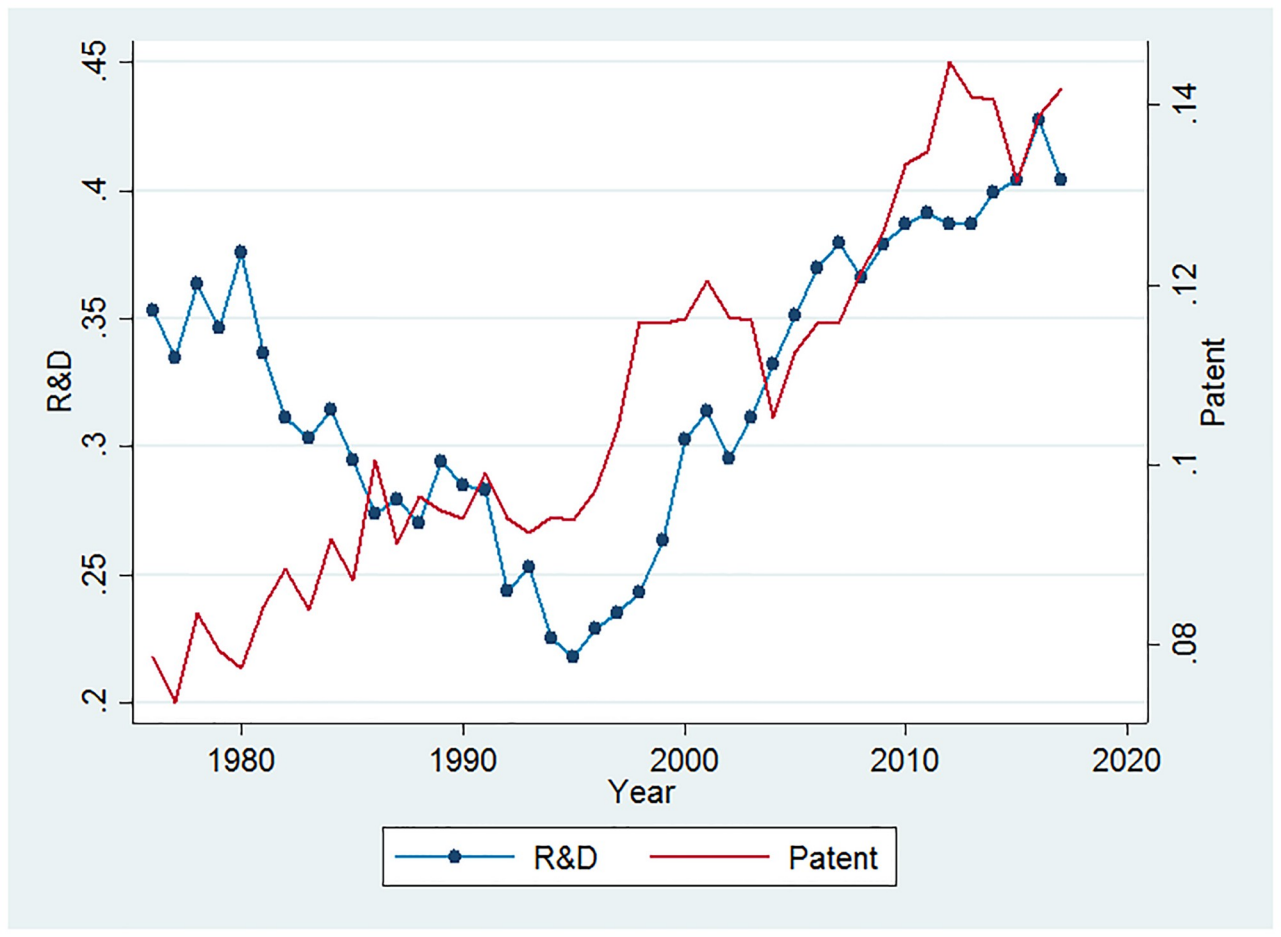
All industries.

**Fig 3:**
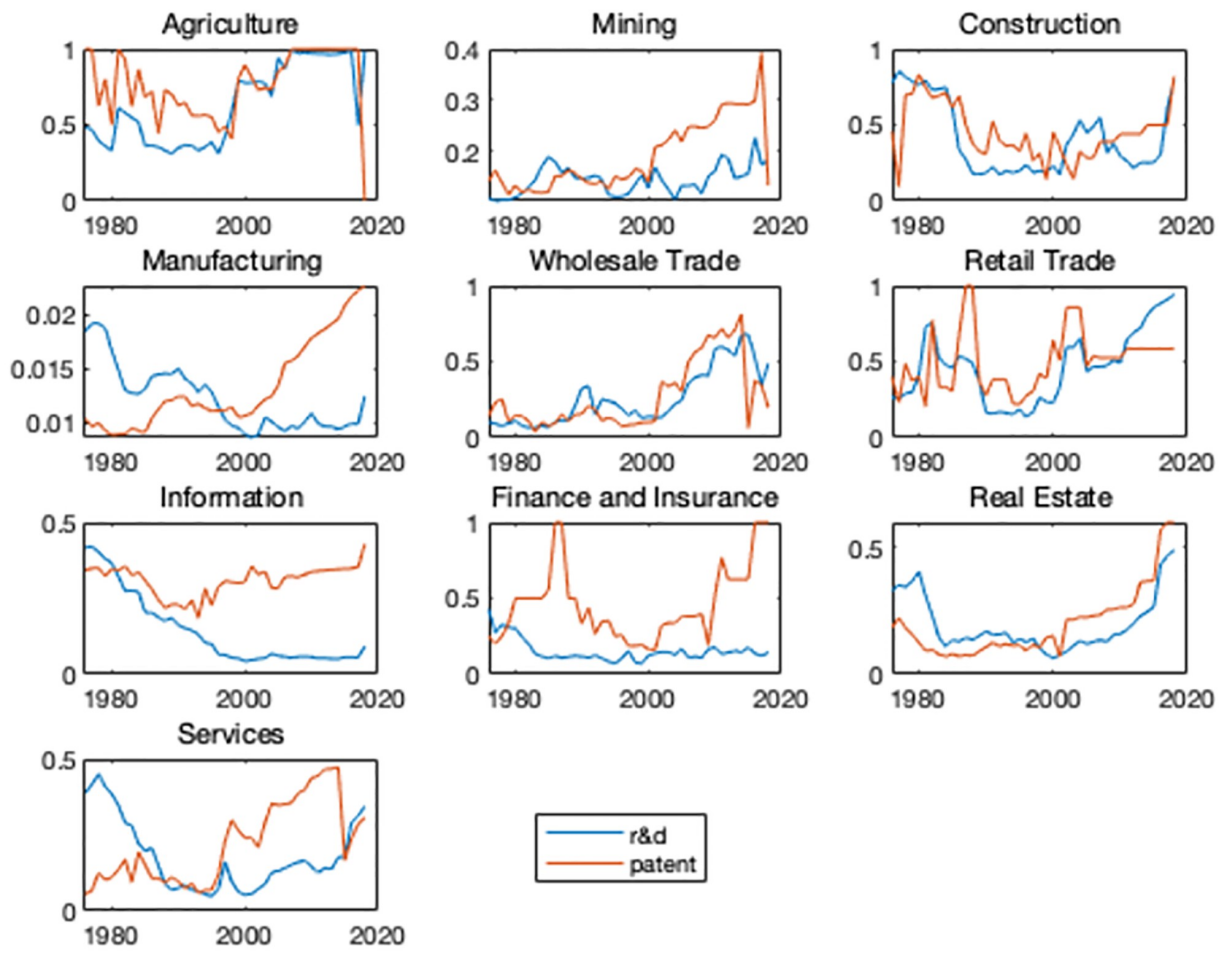
Industry-level HHI of R&D and patent.

In this section, I examine the industry-level changes in HHI index. I construct both R&D HHI and patent HHI as two measures of industrial innovation concentration. To be able to compare the changes across industries with different levels of concentration ratio, for every industry, I calculate HHI index over the entire sample period. In order to have an overall impression, I first look at the HHI in the entire economy. Fig 2 plots the average R&D- and patent-based measures of concentration of all the industries. As said before, 1998–2000 are the years that the number of firms started to decline. I use 1995 as our cutoff since it’s a turning point in industrial concentration. There are clear upward trends over time for both HHI. To check whether the trend exists in all the industries, Fig 3 (detail in appendix) plots both the R&D- and patent-based HHI across all the broad industries. For most of the industries and for both of the two measures of HHI, the industry concentration shows a significant upward trend after 2000. Consistent with our hypothesis, for most of the industries, the trends of the two measures are basically coincident. For industries with relatively large weights, such as manufacturing and information, no obvious upward trend was observed for the R&D based HHI. This is because the transformation and upgrading of traditional large-scale manufacturing firms are difficult and slow, and it is difficult to transform quickly when the economic situation changes, so the profit margin has been maintained at a moderate level. So that manufacturing firms have not formed enough monopoly power. Overall, the general trend of increase in industry concentration is evident.

**Fig 4:**
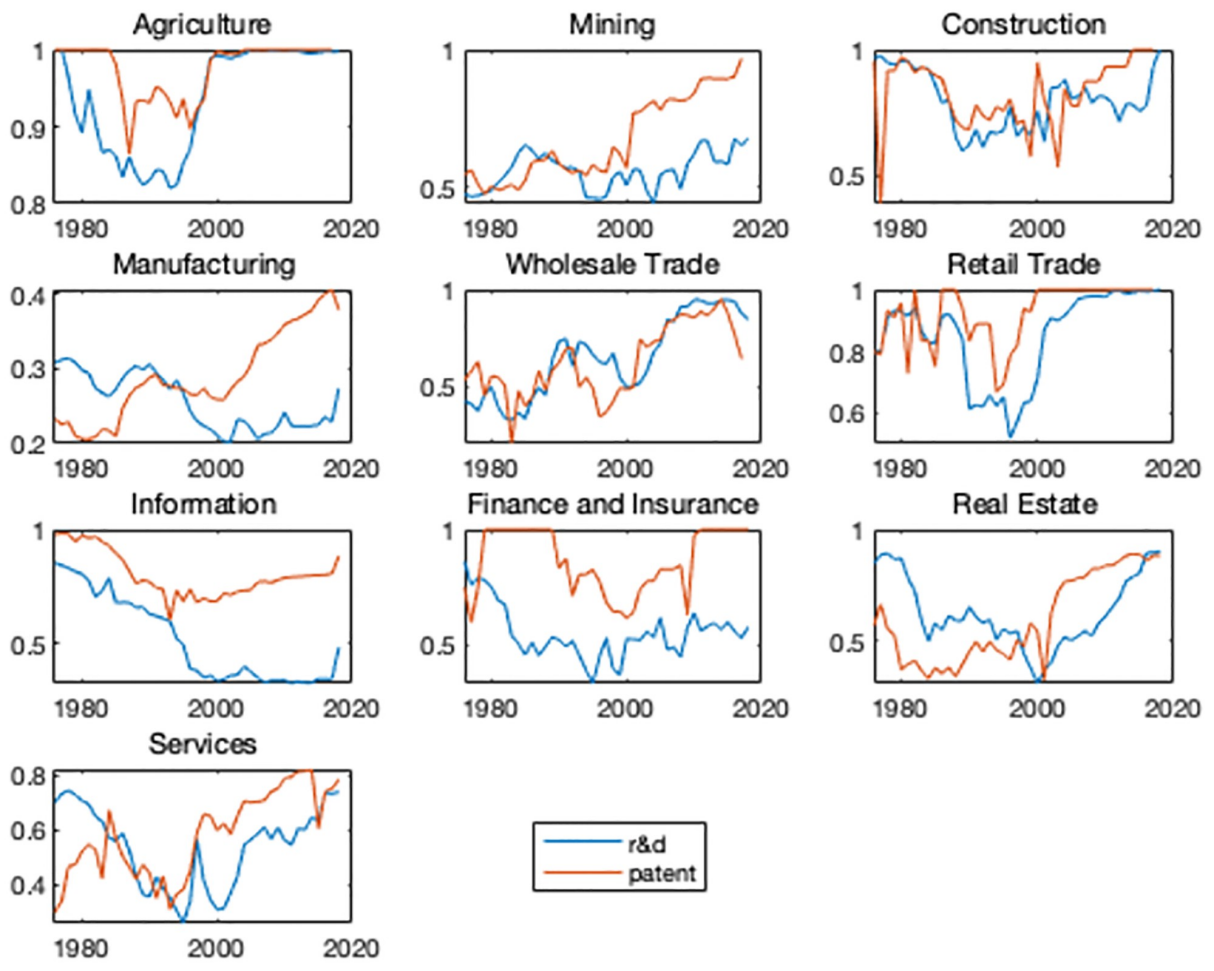
Share of the top 3 firms.

One interesting question is whether the evolution of U.S. innovation activities is dominated by the relative importance of large firms in the economy. Alternatively, whether the increases in concentration results from the R&D and patent share by large firms in the industry started to escalate around 2000. Fig 4 plots the size share of top 3 firms for all the broad industries except for manufacturing which plots the size share of top 10 firms. The share is calculated over the sample period from the Compustat data and NBER patent database. Blue lines show the R&D share calculated as the ratio of the R&D expenditure of the top 3 firms to the specific-industry aggregate R&D expenditure. Yellow lines show the patent share calculated as the ratio of the the top 3 firms’ number of patent to the specific-industry aggregate patent number. Figs 3 and 4 show a high degree of similarity and indicate that innovation activities are gathering in large firms. Since the importance of manufacturing industries in the overall economy has been also declining over the past several decades, I ensure that the increase in concentration is prevalent across the U.S. economy when we look beyond the manufacturing sector.

### Regulation: Entry and exit

There are many potential economic forces that may have contributed to the wide-spread increase in concentration over the last two decades. We identify two important underlying factors: strengthen entry regulation and lax enforcement of antitrust laws. Our evidence suggests that both in their own unique way may have contributed to the increased concentration and barriers to entry.

Barriers to entry have been increasing over the recent years. Entry costs have increased for a variety of reasons, most prominently changes in technology and regulation. [11, 25] demonstrate that entry regulation turns out to be important to explain the rise of entry costs and the decline in the level of entry. Therefore, we use regulation as our proxy for entry costs. The data source about entry regulation is from RegData US 3.1 Annual which aims to measure regulatory stringency at the industry-level. RegData is a text analysis database introduced in [26].

In the United States, antitrust law is a collection of federal and state government laws that regulates the conduct and organization of business corporations, generally to promote competition for the benefit of consumers. The main statutes are the Sherman Act of 1890, the Clayton Act of 1914 and the Federal Trade Commission Act of 1914. These Acts serve three major functions. First, Section 1 of the Sherman Act prohibits price-fixing and the operation of cartels, and prohibits other collusive practices that unreasonably restrain trade. Second, Section 7 of the Clayton Act restricts the mergers and acquisitions of organizations that would likely substantially lessen competition. Third, Section 2 of the Sherman Act outlaws monopolization, attempts to monopolize, and conspiracies to monopolize “any part of the trade or commerce among the several States, or with foreign nations”. We focus on Section 2 of the Sherman Act and Section 7 of the Clayton Act because they prohibits the formation or the abuse of monopoly power. The data source of antitrust enforcement is from the Department of Justice (DoJ).

**Fig 5:**
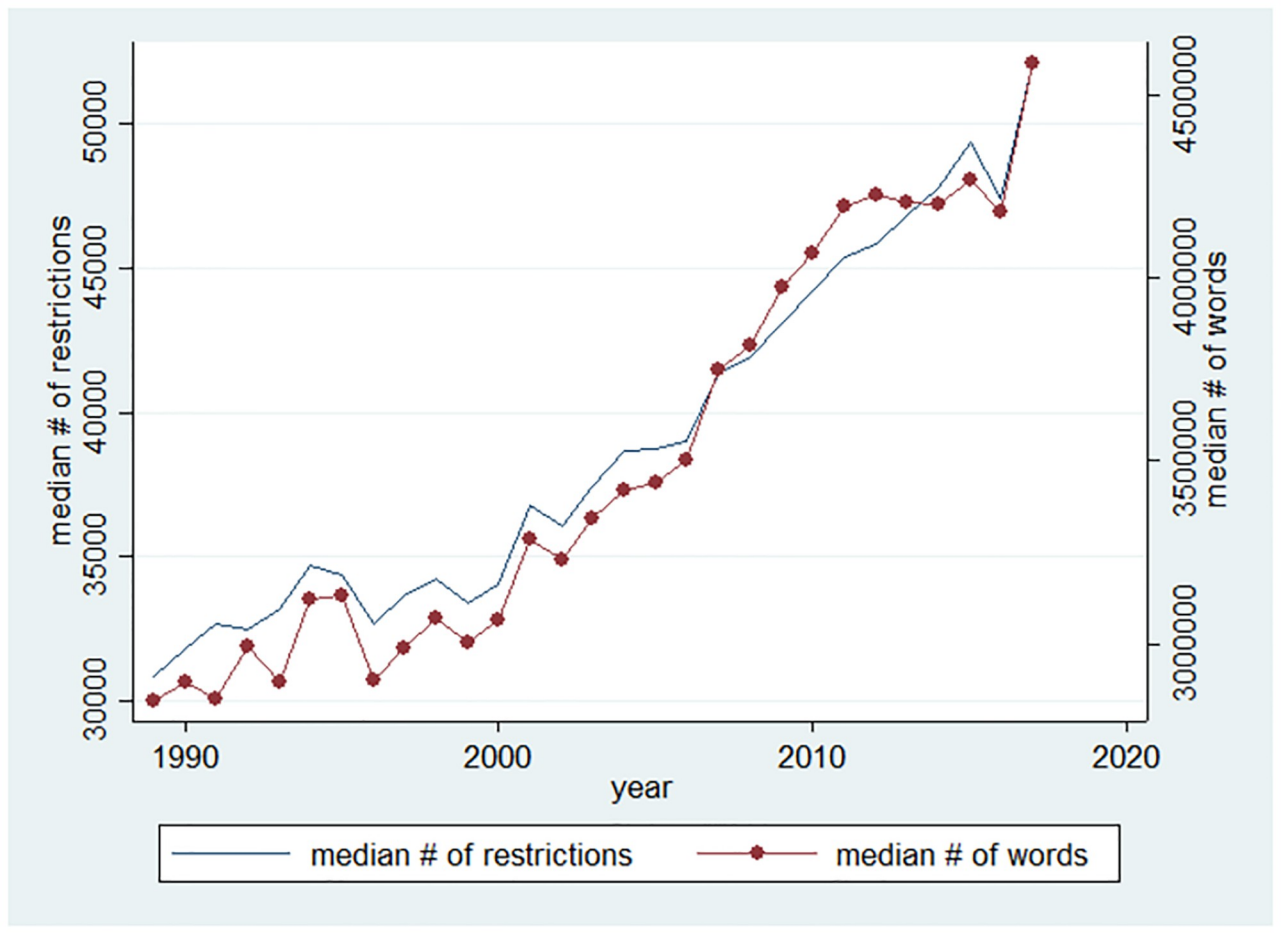
Entry regulation indices.

Fig 5 is consistent with the conjecture that the rise in industry entry costs reduce the number of innovating firms. Comparing Figs 1 and 5, entry costs are significantly increasing since 1999 while the number of innovating firms is noticeably declining. Firms face a higher entry cost while deciding on whether or not to innovate into specific industry. Intuitively, regulations will have strong negative impact on small firms relative to large ones. Small business firms’ profits are not enough to offset the negative impact of entry costs. We will officially explain the proposed hypothesis in our model below.

**Fig 6:**
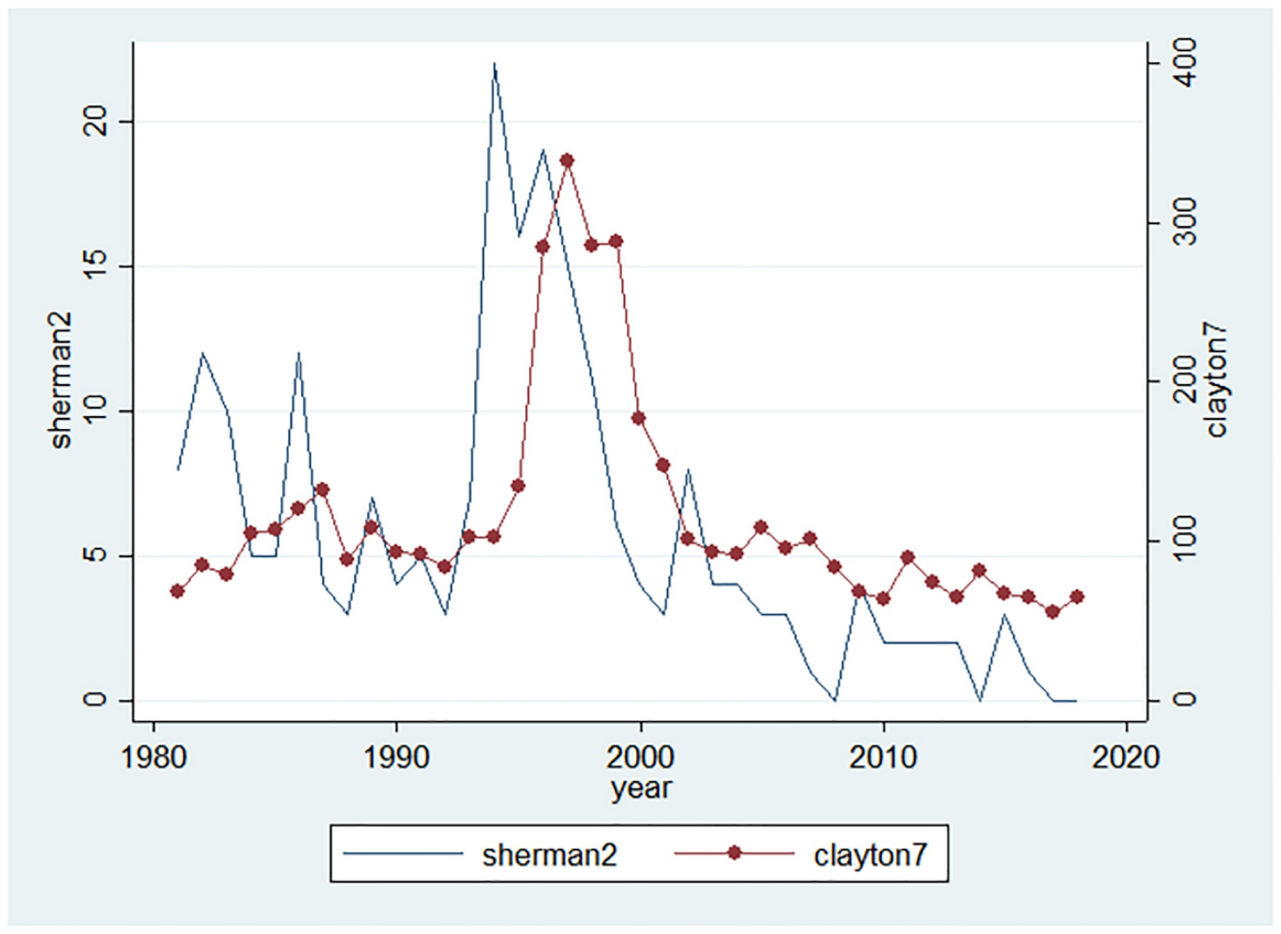
Antitrust enforcement indices.

Fig 6, on the other hand, shows decreasing enforcement of antitrust laws in the late 1990s. The timing is also consistent with our observation in Fig 3 that industry-level concentration of R&D and patent start to escalate. The decline in the number of firms and R&D are relatively concentrated in large firms are two important aspects of the increase in industrial concentration, thus increase in entry costs driven by regulation and decrease in antitrust enforcement are plausible explanations for the increase of concentration in most industries.

### Fact 3. Decrease in firm concentration

Another interesting question is whether these increases in concentration are mainly due to firms expanding their scope over multiple industries, or rather are due to firms focus on core industries. To address this question, I calculate different firm’s dynamic decision of entry and exit across industries. Generally, a firm will conduct research and create knowledge/patents in different sectors over time. Firms’ distributions of patents in different sectors will also change over time. To investigate how intensive and extensive firms innovate in different sectors, I construct firms’ patent concentration index in two steps using NBER patent data. First, I calculate the patent concentration of each firm. The calculation methodology is: $\sum_{i=1}^{N}{\left[\frac{Z(i,k)}{\sum_{i=1}^{N}Z(i,k)}\right]}^{2}$. Where *Z*(*i*, *k*) represents the amount of patents firm *k* owns in sector *i*. I call the patent concentration *firm*-*levelHHI* here.

**Fig 7:**
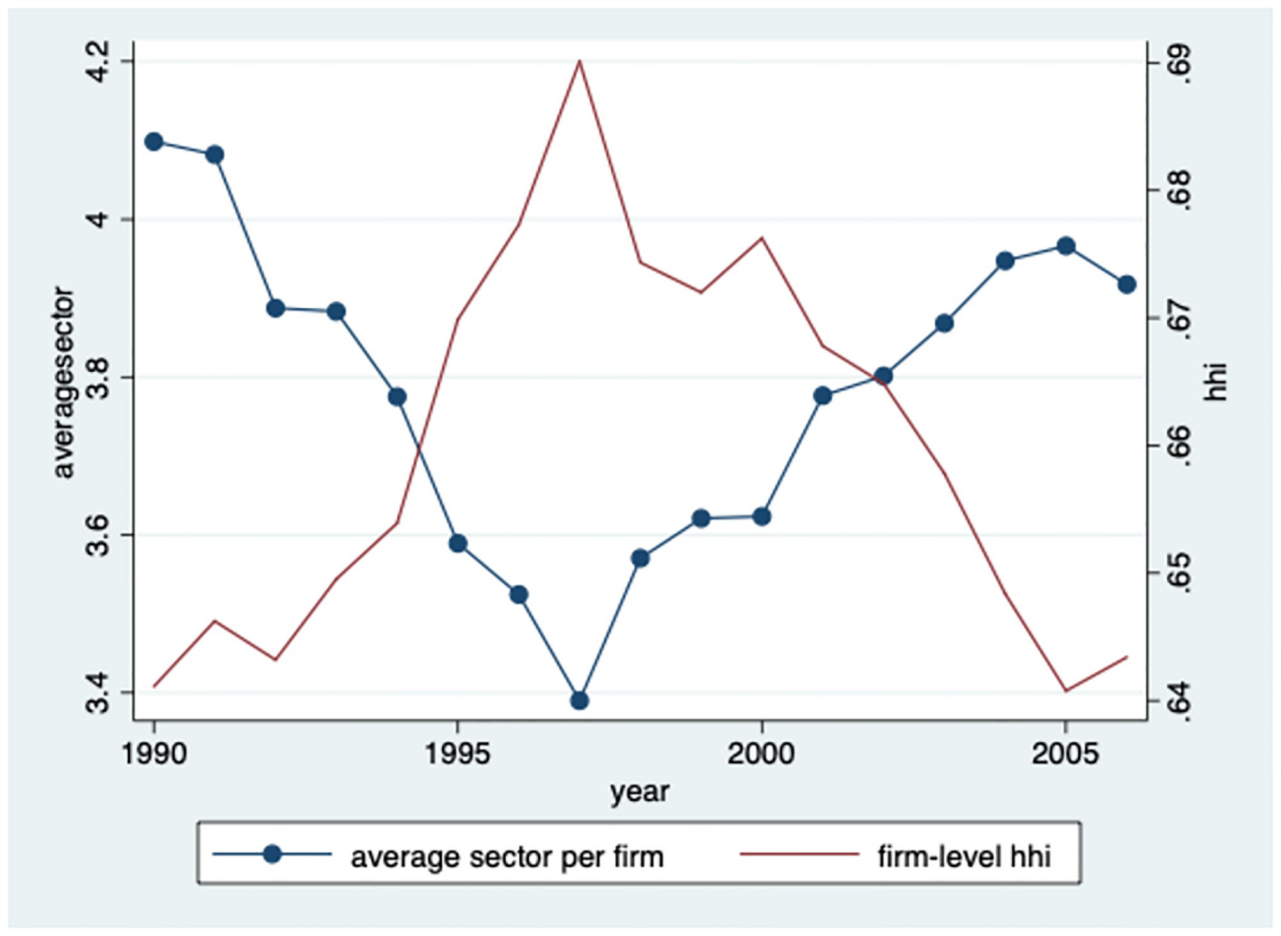
Firm-level concentration.

Next, the patent concentration of all firms (*firm*-*levelHHI*) are averaged by the total number of firms within every year. Fig 7 illustrates firms’ patent concentration index (red line) using NBER patent data in 1990–2006. This *firm*-*levelHHI* shows a clear downward trend since 1997, indicating that firms expand their scope over multiple industries. I also divided firms into two categories, one is that the number of patents is more than 100, and the other is less than 100. The results are consistent and as shown in Table 2 that bigger firms enter more sectors.

**Table 2:** Firm size and annual change of sectors.

VARIABLES	annual change
patent number	0.7187*** (0.2765)
Observations	50,793

Standard errors in parentheses

*** p<0.01,

** p<0.05,

* p<0.1

Note: This table reports regression results of the annual growth rate of sectors that firms enter on patent number which is a measure of patent market share. The data is based on NBER patent data project and USPTO. Measure of growth rates are standard. Standard errors are clustered at the industry level in parentheses.

There are two opposing forces to influence patent concentration: Ceteris paribus, innovate into more industries will decrease firm-level HHI and conduct research/R&D relatively more into specific sectors will increase firm-level HHI. Obviously, this figure shows that the former dominates the latter. Firms allocate R&D resources to new sectors tend to decrease R&D resources of existing sectors, which in turn decrease firm-level HHI. Although firms invest intensively in R&D in the existing “central” sectors, the extensive margin dominates the intensive margin, which decreases the firm-level concentration.

### Fact 4. Similar trends between R&D and product markets

**Fig 8:**
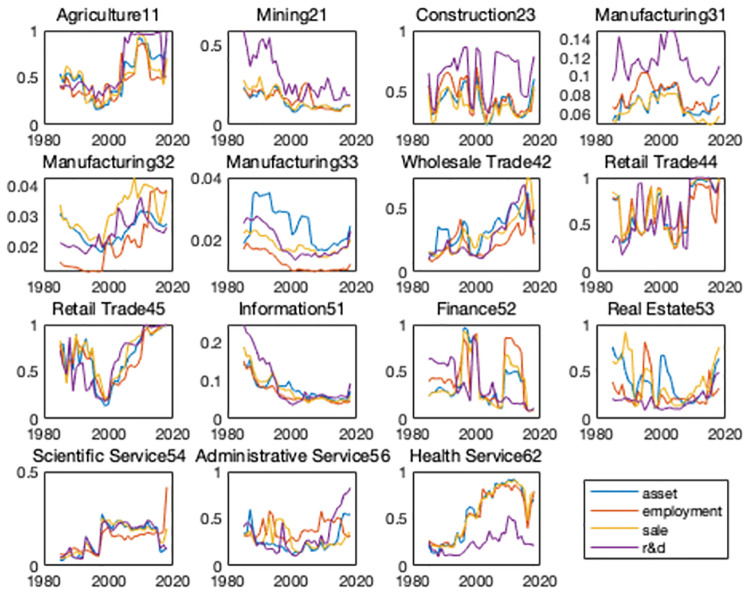
Industry-level HHI of product and R&D.

I use asset, employee and sale to measure the performance of the companies in the product market, and use R&D to measure the performance of the companies in the R&D market. I construct the HHI within every NAICS 2-digit industry-year during 1985–2017 as before. Fig 8 plots industry-level HHI of product and R&D markets. On one hand, the high degree of consistency across the three different proxy variables indicates that they are good measures of the concentration index of the product market. On the other hand, the high degree of consistency between product and R&D proxy variables shows that company’s behavior in product and R&D is highly consistent.

This is not surprising since innovating firms could create blueprints for new varieties and manufacture the products that have been invented at the same time, I bridge the gap between the product market and the R&D market in the model below. Intuitively, both markets would have similar trends in industry concentration. As interpreted in our model, on the one hand, large firms in the R&D market are still large firms in the product market, and large firms in the product market would charge higher price on their product so that they can charge higher profit which is a part of the value of their knowledge on the other hand. Most of the existing literature pay attention to either product market or R&D market concentration, I bridge the gap between them.

### The measurement of technology applicability

#### Calculating sector-specific technology applicability

For simplicity, I use the citation count (or variation of it such as Garfield’s (1972) “impact factor” or the forward-citation-weighted count) as measure of sector-specific technology applicability. More precisely, sector-specific technology applicability is calculated as the ratio of sector’s patent citations to total patent citations. [27] construct a measure quantifying the applicability of each technology–called “authority weight” in Kleinberg’s original work–which is proved to be the most efficient at extracting information from a highly linked knowledge diffusion network. They also construct three alternative measure to rank sectors’ technology applicability, which include the method used in this paper. It turns out that the four measures of technology applicability are highly correlated with each other and their emprical results are robust to using alternative measures. Without loss of generality, I use the simplist but efficient method to measure technology applicability.

#### Calculating firm’s technology applicability

Each firm is identified by its overall patent stock (*Z*_*f*,*t*_), and its technological position (*P*_*f*,*t*_). Following Jaffe (1986), I characterize the firm’s technological position by the distribution of its patent over all patent subcatgories, defining a vector $P_{f,t}=(P_{f,t}^{1},\cdots ,P_{f,t}^{i},\cdots ,P_{f,t}^{37})$ where $P_{f,t}^{i}$ is the share of patents of firm f in technology subcatgory *i* at time *t*. This vector also characterizes the firm’s knowledge distribution. A firm’s overall technology applicability measure, *TA*_*f*,*t*_, is calculated as the weighted geometric mean of the applicability of its technologies: $TA_{f,t}=\prod_{i\in j}{\left(a^{i}\right)}^{P_{f,t}^{i}}$. *a*^*i*^ is sector *i*’s technology applicability. To measure multi-technology patenting (or technology scope), I count the number of distinct technology subcatgories in which firm has patented.

#### Identification strategy

$$\frac{sale_{t}^{i}}{sale_{t}}=\beta_{1}share_{t}^{i}+\beta_{2}TA_{t}^{i}+d_{t}+\epsilon_{i,t}$$

$\frac{sale_{t}^{i}}{sale_{t}}$ is the sales share of sector *i* at time *t*. $share_{t}^{i}$ is the sector *i*’s share of patents. *β*_1_ > 0 implies that a larger share of sector’s patents obtain higher sales in this sector. *β*_2_ > 0 implies on average a highly applicable sector make bigger sales.

Table 3 is sector-level evidence about the significantly positive relationship between sales and patent shares. It’s consistent with Fig 8 which plots industry-level HHI of product and R&D markets. This regression result not only provides data support for the relationship at the sector level, but also provides a basis for the connection of the two shares at the firm level. And I will show you firm-level evidence next.

**Table 3:** Industry level market share of patents and sales.

VARIABLES	sector share of sales
sector share of patents	.728*** (.029)
technology applicability	.167*** (.0238)
constant	.0028*** (.00034)
Observations	1,138
R-squared	0.8619

Standard errors in parentheses

*** p<0.01,

** p<0.05,

* p<0.1

Note: This table reports regression results of the sector share of sales on sector share of patents controlling for sector-specific technology applicability and time effects. As indicated in this table, sector share of patents shows a significantly positive relationship with sector share of sales. This strengthens the view that R&D and product markets have similar trends.

#### Identification strategy

$$\frac{sale_{k,t}^{i}}{sale_{t}^{i}}=\beta_{1}share_{k,t}^{i}+\beta_{2}TA_{k,t}+d_{k}+d_{t}+\epsilon_{i,t}$$

$sale_{k,t}^{i}$ is sales of firm *k* in sector *i* at time *t*. $sale_{t}^{i}$ is sector *i*’s total sales. $share_{k,t}^{i}$ is firm *k*’s share of patents in sector *i* at time *t*. *TA*_*k*,*t*_ is firm *k*’s technology applicability at time *t*. *d*_*k*_ and *d*_*t*_ are firm and year fixed effects.

#### Firm-level evidence

Table 4 shows that firm’s share of sales and its share of patents are significantly positive controlling for firm’s technology applicability. This suggests that the industry level similar trends between R&D and product markets is derived from firm level trends in the two markets. Furthermore, we test whether the coefficient is 1 or not. Consistent with **Fact 4**, the test result does NOT reject the null hypothesis at the 1% significance level.

**Table 4:** Firm level market share of sales and patents.

VARIABLES	firm’s share of sales
firm’s share of patents	1.012*** (.0479)
technology applicability	.00679*** (.000578)
constant	-0.00331*** (.000602)
Observations	147,367
R-squared	0.421

Robust standard errors in parentheses

*** p<0.01,

** p<0.05,

* p<0.1

Note: This table reports regression results of the firm’s share of sales on firm’s share of patents. This table shows that firm level patents share and firm level sales share are significantly positive controlling for firm’s technology applicability and time effects. This suggests that the industry level similar trends between R&D and product markets is derived from firm level trends in the two markets. The data is based on NBER patent data project and USPTO and Compustat merged data. Robust standard errors are clustered at the industry level in parentheses.

## Model

This section spells out the model environment. There are two types of agents: households and firms. I describe the structure of the economy and solve for the households and firms’ problem. First, there is a continuum of households each of whom consumes and supplies labor. Second, a finite number of firms distributed in an industry, and create a single differentiated variety. The number of industries is exogenous and equal to *N*. Firms set their price and quanyity strategically in industries which are linked by a knowledge network.

### Household

The households in the model are homogenous with a unit mass. The representative household maximizes utility:

$$\begin{array}{c} U\left(C_{1},\cdots ,C_{N}\right)=\theta \prod_{i=1}^{N}C_{i}^{\beta_{i}} \end{array}$$ (1)

where *C*_*i*_ represents composite consumption of varieties from industry *i* and *θ* is a normalization constant, $\theta =\prod_{i=1}^{N}\beta_{i}^{-\beta_{i}}$. The Cobb-Douglas weights *β*_*i*_ determine household tastes for goods and services from different industries, which are equal to the expenditure shares of each good $\frac{P_{i}C_{i}}{P_{c}C}$. *P*_*i*_ is the price of good *i* and *P*_*c*_ is the aggregate price index that satisfies $P_{c}=\prod_{i=1}^{N}P_{i}^{\beta_{i}}$.

The composite consumption good produced by industry *i* is given by:

$$\begin{array}{c} C_{i}={\left(\sum_{k=1}^{N_{i}}C{\left(i,k\right)}^{\frac{\varepsilon_{i}-1}{\varepsilon_{i}}}\right)}^{\frac{\varepsilon_{i}}{\varepsilon_{i}-1}} \end{array}$$ (2)

where *C*(*i*, *k*) is household consumption from firm *k* in industry *i* and *ε*_*i*_ > 1 is the elasticity of substitution across firms within industry *i*. When *ε*_*i*_ → ∞, we recover the case where varieties are perfect substitutes, and there is no product differentiation. An integer number, *N*_*i*_, of varieties exists in industry *i*. Finally, the price of good *i* satisfies:

$$\begin{array}{c} P_{i}={\left(\sum_{k=1}^{N_{i}}P{\left(i,k\right)}^{1-\varepsilon_{i}}\right)}^{\frac{1}{1-\varepsilon_{i}}} \end{array}$$ (3)

where *P*(*i*, *k*) is the price of variety *k* in sector *i*. Each variety is produced by exactly one firm, and all the firms are owned by the representative household.

### Production

Firms undertake two distinct activities. They can not only create blueprints for new varieties but also manufacture the products that have been invented. In other words, firms can use part of their patents for research and development and the rest for producing products. The firm inventing a new variety is the unique supplier of that variety. For simplification, I assume that each differentiated good is manufactured according to a common technology: to produce one unit of any product requires one unit labor and one unit blueprint.

## Innovation

The firm *k* in sector *i* combines labor, *l*(*i*, *k*), and other sectors’ blueprints, *Z*(*i*, *k*, *j*), to create *Z*(*i*, *k*) units of its variety using the Cobb-Douglas technology:

$$\begin{array}{c} Z\left(i,k\right)=\alpha_{i}\varphi \left(i,k\right)l{\left(i,k\right)}^{r_{i}}\prod_{j=1}^{N}Z{\left(i,k,j\right)}^{\omega_{ij}} \end{array}$$ (4)

where *r*_*i*_ is the labor share in the knowledge creation function, *α*_*i*_ is a normalization constant, which makes the mathematics simpler and is equal to $\alpha_{i}=r_{i}^{-r_{i}}\prod_{j=1}^{N}\omega_{ij}^{-\omega_{ij}}$. *φ*(*i*, *k*) is firm-specific productivity to the firm *k* in sector *i* and it’s Hicks-neutral efficiency, *ω*_*ij*_ is the input share of sector *j*’s blueprints needed in sector *i*’s knowledge creation process. Ω = {*ω*_*ij*_}_*i*,*j*_ is a (*N* × *N*) matrix which represents the knowledge network.

Thanks to constant return-to-scale, the *i*th rows of *ω* sum to $1-r_{i}:\sum_{j=1}^{N}\omega_{ij}=1-r_{i}$. Fuethermore, *Z*(*i*, *k*, *j*) is a composite of sector *j*’s varieties such that:

$$\begin{array}{c} Z\left(i,k,j\right)={\left\{\sum_{l=1}^{N_{j}}Z{\left(i,k,j,l\right)}^{\frac{\varepsilon_{j}-1}{\varepsilon_{j}}}\right\}}^{\frac{\varepsilon_{j}}{\varepsilon_{j}-1}} \end{array}$$ (5)

where *Z*(*i*, *k*, *j*, *l*) is the quantity of the variety *l* of sector *j*’s blueprints that is used for the knowledge creation of variety *k* of sector *i*’s knowledge. Note that the elasticity of substitution among varieties in a sector is the same for firms and for the household.

A sector is also defined as a market in which firms are engaged in imperfectly competition. Sector’s knowledge is imperfectly substitute and varieties within a sector are more substitute: *ε*_*i*_ > 1. Each firm creates exactly one variety, and consumers cannot perfectly substitute between two varieties: *ε*_*i*_ < ∞. Following [9], I assume firms play a static game where firm *k* in sector *i* chooses its price *P*(*i*, *k*) taking as given the prices chosen by other firms in the economy, the other sectors’ price and quantities, the wage, and aggregated prices and quantities. Importantly, note that this firm recognizes that sector *i*’s price and quantity are affected when it changes its price.

Solve the cost minimization problem, firm *k* in sector *i* faces a marginal cost:

$$\begin{array}{c} \lambda \left(i,k\right)=\varphi {\left(i,k\right)}^{-1}w^{r_{i}}\prod_{j=1}^{N}P_{j}^{\omega_{ij}} \end{array}$$ (6)

where *w* is the wage rate in this economy. Note that due to the presence of knowledge linkages, this marginal cost is a function of other sectors’ prices. The higher the productivity of the firm, the lower the marginal cost. The sector-level gross knowledge is defined as:

$$\begin{array}{c} Z_{i}={\left\{\sum_{k=1}^{N_{i}}Z{\left(i,k\right)}^{\frac{\varepsilon_{i}-1}{\varepsilon_{i}}}\right\}}^{\frac{\varepsilon_{i}}{\varepsilon_{i}-1}} \end{array}$$ (7)

Formally, I characterize the pricing decision of a firm *k* in sector *i* as follows: I assume that the individual patents developing firms are engaged in imperfectly competition. In most of the results that follow, I take as a baseline case a model of imperfectly competition based on the assumption that firms play a static game of quantity competition. Specifically, each firm *k* chooses its patent quantity *Z*(*i*, *k*) sold in industry *i* taking as given the quantities chosen by the other firms in the economy, as well as the aggregate patent price *P* and quantity *Z*. Note that under this assumption, each firm does recognize that sectoral theoretical price index *P*_*i*_ and quantity index *Z*_*i*_ vary when that firm changes its quantity *Z*(*i*, *k*).

I solve the firm’s pricing problem under the assumptions above as follows. Putting the assumptions and profit maximization conditions together allows us to give the following characterization of innovating firm’s pricing strategy in term of endogenous variable markup.

**Proposition 1** *Firm k in sector i sets a price P(i, k), a markup μ(i, k) and has a patent share s(i, k) that satisfies the following system of equations*:

$$\begin{array}{c} P\left(i,k\right)=\lambda \left(i,k\right)\mu \left(i,k\right) \end{array}$$ (8)

$$\begin{array}{c} \mu \left(i,k\right)=\frac{\varepsilon_{i}}{\varepsilon_{i}-1-\left(\varepsilon_{i}-1\right)s\left(i,k\right)} \end{array}$$ (9)

$$\begin{array}{c} s\left(i,k\right)=\frac{P\left(i,k\right)Z\left(i,k\right)}{P_{i}Z_{i}}={\left(\frac{P\left(i,k\right)}{P_{i}}\right)}^{1-\varepsilon_{i}} \end{array}$$ (10)

The first thing to note in the above proposition is that firms charge a markup over their marginal cost λ(*i*, *k*). The markup charged is increasing in the patent share of the firm: larger firms charge a higher markup. When $s\left(i,k\right)\to 0,\;\mu \left(i,k\right)\to \frac{\varepsilon_{i}}{\varepsilon_{i}-1}$. That is to say, as a firm becomes atomistic, its markup approaches the one under monopolistic competition.

**Corollary 1**: *Firm with higher productivity sets a lower marginal cost, higher markup, lower price, and obtains higher market share*.

From the corollary above, we can know that the productivity of a firm completely determines its market share.

### Sector-level markup

An important variable is the sector-level markup. This markup is defined as the sector-level price divided by the sector-level marginal cost. For a given sector *i*, the marginal cost is defined as $\lambda_{i}=\frac{dTC_{i}}{dZ_{i}}$, where *TC*_*i*_ is the total cost in sector *i*: $TC_{i}=\sum_{k=1}^{M^{i}}\lambda \left(i,k\right)Z\left(i,k\right)$. Note that in the context of constant return-to-scale, the marginal cost is also equal to the average cost; therefore, $\lambda_{i}=\frac{TC_{i}}{Z_{i}}=\sum_{k=1}^{M^{i}}\lambda \left(i,k\right)\frac{Z\left(i,k\right)}{Z_{i}}$. After using the fact that firm-level price is a markup over the marginal cost, it is easy to see the sector-level markup *μ*_*i*_ is

$$\begin{array}{c} \mu_{i}=\frac{P_{i}}{\lambda_{i}}={\left(\sum_{k=1}^{M^{i}}\mu {\left(i,k\right)}^{-1}s\left(i,k\right)\right)}^{-1} \end{array}$$ (11)

The sector’s markup is a patent-share-weighted harmonic average of firm-level markups. This expression is valid as long as firms charge a markup over the marginal cost. Lemma 1 below shows the sector-level markup is a function of the sector-level concentration index. In particular, the directly observable Herfindahl-Hirchman-Index (HHI), the sum of the patent share squared, plays an important role.

**Lemma 1 (Sector-Level Markup)** *The sector i*′ *s markup is equal to* $\mu_{i}=\frac{\varepsilon_{i}}{\varepsilon_{i}-1}{\left(1-HHI_{i}\right)}^{-1}$ .

Where $HHI_{i}=\sum_{k=1}^{M^{i}}s{\left(i,k\right)}^{2}$ is the sector *i*′*s* Herfindahl-Hirchman-Index.

The above lemma shows that under imperfectly competition, the HHI entirely determines the sector’s markup. The intuition is as follows: when the sector’s concentration is high, that is when the HHI is high, large firms have a higher market share and thus they can use this higher market power to charge higher markups, which in turn aggregate to a higher sector’s markup. An important implication of Lemma 1 is that it links empirically observable variables, such as the HHI, to the sector-level markup. Using the result in the above lemma, it is easy to derive that a higher sector’s HHI always implies a higher sector’s markup. In this framework, given the demand system and the assumed market structure, sector concentration is a measure of sector competition.

The other important variable to define is the sector-level productivity. As shown above, the sector-level marginal cost is $\lambda_{i}=\sum_{k=1}^{N_{i}}\lambda \left(i,k\right)\frac{Z\left(i,k\right)}{Z_{i}}$. After substituting for the firm-level marginal cost $\lambda \left(i,k\right)=\varphi {\left(i,k\right)}^{-1}w^{r_{i}}\prod_{j=1}^{N}P_{j}^{\omega_{ij}}$, the sector-level marginal cost is equal to $\lambda_{i}=\varphi_{i}^{-1}w^{r_{i}}\prod_{j=1}^{N}P_{j}^{\omega_{ij}}$, where $\varphi_{i}^{-1}=\sum_{k=1}^{N_{i}}\varphi {\left(i,k\right)}^{-1}\frac{Z\left(i,k\right)}{Z_{i}}$ is the sector *i*’s productivity. It is entirely determined by the joint distribution of patent share and productivities across firms in a sector, whereas the sector-level markup is entirely determined by the distribution of patent share.

### Equilibrium

**Definition 1** *A general equilibrium is a collection of prices p(i, k), wage w, and input demands Z(i, k, j, l), newly-created knowledge Z(i, k), consumption C(i, k) and labor demands l(i, k) such that for given vectors of productivity*:

*(i) Each firm minimizes its costs subject to demand function*,

*(ii) the representative household chooses consumption to miximize utility*,

*(iii) markets for each patent and labor clear*.

Similarly to [28], I introduce a notion of centrality, the supplier centrality, and the consumer centrality is always equal to one for every industry in our Cobb-Douglas case.

**Definition 2** *The supplier centrality is*

$$\begin{array}{c} {\tilde{\beta }}^{\prime }=\beta^{\prime }{\left(I-\tilde{\Omega }\right)}^{-1} \end{array}$$ (12)

*where*

$$\begin{array}{c} \tilde{\Omega }={\left\{\mu_{i}^{-1}\omega_{ij}\right\}}_{1\leq i,j\leq N} \end{array}$$ (13)

In this framework, the supplier centrality is a key factor since it determines the size distribution of industries in the economy. It represents the importance of each industry on the supply side.

**Proposition 2**

$$\begin{array}{c} {\left\{logP_{i}\right\}}_{i}={\left(I-\Omega \right)}^{-1}{\left\{log\mu_{i}\varphi_{i}^{-1}w^{r_{i}}\right\}}_{i} \end{array}$$ (14)

*where I is the (N × N) identity matrix*.

*Sectors’ patent shares are equal to*:

$$\begin{array}{c} {\left\{\frac{P_{i}Z_{i}}{P_{c}C}\right\}}_{i}^{\prime }=\beta^{\prime }{\left(I-\tilde{\Omega }\right)}^{-1}={\tilde{\beta }}^{\prime } \end{array}$$ (15)

*where the (N × N) matrix* $\tilde{\Omega }$ *is such that* $\tilde{\Omega }={\left\{\mu_{i}^{-1}\omega_{ij}\right\}}_{1\leq i,j\leq N}$ *and the (N × 1) vector β is such that β = {β_i_}_i_*. ${\tilde{\beta }}^{\prime }$ *is called supplier centrality. We can think of* ${\tilde{\beta }}^{\prime }$ *as the network-adjusted consumption share of the industries. The ith element of* ${\tilde{\beta }}^{\prime }$ *captures demand from the household that reaches industry i, whether directly or indirectly through other industries who use i’s patents. In other words, supplier centrality captures the importance of the industry as a supplier to the household*.

The above proposition characterizes the sectors’ allocation for a given wage. System (14) of *N* equations relates the sectors’ prices to sectors’ productivities *φ*_*i*_, sectors’ markups *μ*_*i*_, wage *w*, and the knowledge network Ω.

A key result of this paper, and one that delivers much of the intuition for the results, is the following characterization of active firms’ profit functions in terms of supplier centrality measure and market share:

**Proposition 3** *The profit of firm k in sector i is equal to*

$$\begin{array}{c} \pi \left(i,k\right)=\frac{\mu \left(i,k\right)-1}{\mu \left(i,k\right)}s\left(i,k\right){\tilde{\beta }}_{i}P_{c}C-\phi_{i} \end{array}$$ (16)

Proposition 3 implies that the profits of a firm are determined by a few intuitive key statistics. The term $\frac{\mu \left(i,k\right)-1}{\mu \left(i,k\right)}$ converts a firm’s sales into profit which is positively correlated with market share *s*(*i*, *k*). ${\tilde{\beta }}_{i}$ is the supply-side centrality of the industry. Finally, we arrive at a firm’s net profits by subtracting the sector-specific fixed costs *ϕ*_*i*_ from its gross profit. This sector-specific cost of innovation can be interpreted as legal barrier to entry or the cost of maintaining a research lab.

From the proposition above, the firm’s profit can be decomposed into three parts: firm-level market share *s*(*i*, *k*), sector-level supplier centrality ${\tilde{\beta }}_{i}$, and aggregate demand *P*_*c*_ *C*. According to corollary 1, firm’s productivity determines market share. Then, given supplier centrality and aggregate demand, firm’s decision on whether to innovate is simply determined by firm’s productivity and fixed cost. As entry costs increase, low productivity and low market share firms exit.

#### Examples

**Fig 9:**
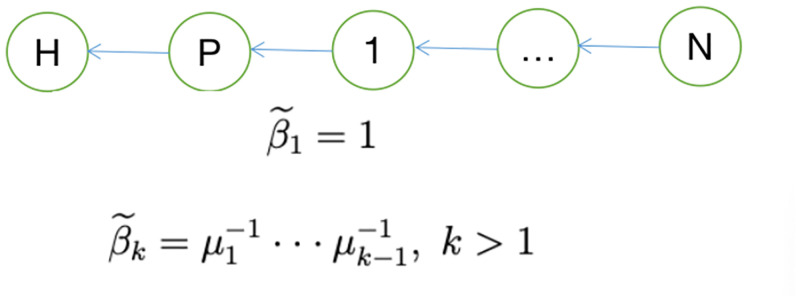
Vertical network.

**Fig 10:**
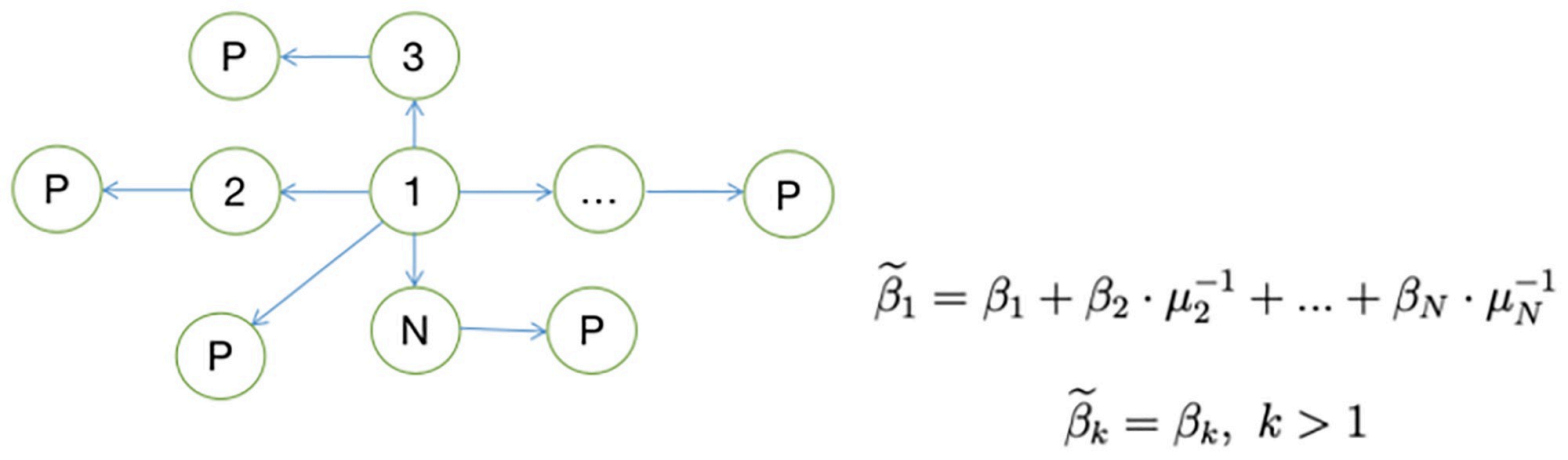
Star network.

In the following examples, I will focus on the supplier centrality of the industry corresponding to different knowledge network structures, and examine two common network structures: vertical network structure and star network structure. I have given the shape of the two network structures in Figs 9 and 10 respectively.

For vertical economies, only sector *N* directly uses labor for research and development, and only sector 1 uses patents in the product market. In a vertical network, *β* = (1, 0, ⋯, 0)′, so we can use the definition of supplier centrality to calculate the supplier centrality of each sector. The supplier centrality of sector 1 is 1, while the supplier centrality of other sectors are less than 1. The closer of the industry to the upstream location, the smaller the supplier centrality, which is consistent with our intuition.

For star economies, only sector 1 directly uses labor for research and development, all other sectors use labor and sector 1’s patents for research and development. All sectors use part of their patents on the product market for production and ultimately provide them to consumers. In a star network, similarly, we can calculate the supplier centrality of each sector. The supplier centrality of sector1 is ${\tilde{\beta }}_{1}=\beta_{1}+\beta_{2}\cdot \mu_{2}^{-1}+\cdots +\beta_{N}\cdot \mu_{N}^{-1}$, and the supplier centrality of other sectors are exactly equal to the consumption share of their sectors respectively. In the absence of a knowledge network: $\Omega =0,\tilde{\beta }=\beta$. All sectors will use all their patents for the product market for production. For such a special case, we can conduct independent research on each sector without considering the interrelationships between sectors.

In the presence of a knowledge network, we can consider the linkages of the concentration levels between sectors. Take the star network as an example, when the concentration of other sectors increase, it means that the markups of these sectors increase. Then it can be inferred from the supplier centrality obtained above that the supplier centrality of sector 1 will decrease. According to Proposition 3, firms with relatively small shares will exit from the market, increasing the concentration level of the sector. In other words, when there is a knowledge network, the concentration trends between sectors have positive linkage effects.

The following proposition shows that as low market share firms exit, sector-level markup, market concentration and sector-level productivity increase.

**Proposition 4** *Without loss of generality, we assume that for any sector i, the vector of firms’ market shares is (s_1_, s_2_, ⋯, s_n_) and s_1_ > s_2_ > ⋯ > s_n_. After fixed costs increase, the vector of firms’ market shares is (s_1_, s_2_, ⋯, s_k_), k < n. We difine* $\mu_{n}=\sum_{i=1}^{n}s_{i}^{2}$ *and* $\mu_{k}=\sum_{i=1}^{k}{\tilde{s}}_{i}^{2}$ , *where* ${\tilde{s}}_{i}$ *is new market share of s_i_ after share adjustment, then μ_n_ < μ_k_. That is, sector-level markup and concentration increased*.

The above proposition proves that due to the rise of fixed cost of R&D, some low-productivity firms begin to withdraw from the market, while high-productivity enterprises continue to occupy a larger market share. For firms with high productivity, there are two forces that increase their market share: one is the exit of small firms, when no new firms enter, then market share of large firms naturally increase; Secondly, firms with high productivity will invest more research and development to occupy a larger market share, further increasing their market share, thus increasing the degree of industry concentration.

To solve for the aggregate output and the equilibrium wage, I assume the household supplies inelastically one unit of labor, and I normalized the price of the composite consumption good to 1. The following proposition describes the equilibrium allocation given sector-level markups and productivities.

**Proposition 5** *For given sector-level markups μ_i_ and productivities φ_i_, the wage is*

$$\begin{array}{c} log\;w=-\beta^{\prime }{\left(I-\Omega \right)}^{-1}{\left\{log\;\mu_{i}\varphi_{i}^{-1}\right\}}_{i} \end{array}$$ (17)

*The share of aggregate profit in nominal output is*

$$\begin{array}{c} \frac{Pro}{P_{c}C}=\beta^{\prime }{\left(I-\tilde{\Omega }\right)}^{-1}{\left\{1-\mu_{i}^{-1}\right\}}_{i}=\sum_{i=1}^{N}\beta_{i}\left(1-{\tilde{\mu }}_{i}^{-1}\right) \end{array}$$ (18)

*where* ${\tilde{\mu }}_{i}$ *is such that* ${\left\{1-{\tilde{\mu }}_{i}^{-1}\right\}}_{i}={\left(I-\tilde{\Omega }\right)}^{-1}{\left\{1-\mu_{i}^{-1}\right\}}_{i}$ . *Finally, aggregate output is*:

$$\begin{array}{c} log\;C=log\left(w-\sum_{i=1}^{N}N_{i}\phi_{i}\right)-log\left(1-\tilde{\beta }{\left\{1-\mu_{i}^{-1}\right\}}_{i}\right) \end{array}$$ (19)

Proposition 5 proves that the share of profit in revenue is only a function of sector-level markup and has nothing to do with sector-level productivity. This is because the productivity change at the sector level is the same for all firms, and does not change the market power among them, so it does not affect the concentration degree and profits at the sector-level. For the equilibrium wage level, it depends not only on the sector-level markup, but also on the sector-level of productivity. If the total-level of consumption is defined as the level of consumers’ welfare, then it is a function of the share of profits and wage. That is, consumers’ welfare depends on the productivity and markup of the specific sector. With explicit expressions or analytic solutions for consumers’ welfare, We’re possible to analyze how the changes in sectoral markups or monopoly levels will affect consumers’ welfare.

### Comparative static analysis

From proposition 5, we can see that the sector-level markup will affect the wage and profit share. The derivative of wage and profit share on the markup *μ*_*i*_ can be obtained as follows:

$$\begin{array}{c} \frac{\partial logw}{\partial log\mu_{i}}=-{\bar{\beta }}_{i}<0 \end{array}$$ (20)

$$\begin{array}{c} \frac{\partial \left(\frac{Pro}{P_{C}C}\right)}{\partial \mu_{i}}=\mu_{i}^{-1}{\tilde{\beta }}_{i}\left[\mu_{i}^{-1}-e_{i}^{{}^{\prime }}\left({\left(I-\tilde{\Omega }\right)}^{-1}-I\right){\left\{1-\mu_{i}^{-1}\right\}}_{i}\right]>0 \end{array}$$ (21)

where $\bar{\beta }{}^{\prime }=\beta^{\prime }{\left(I-\Omega \right)}^{-1}$,$\tilde{\beta }{}^{\prime }=\beta^{\prime }{\left(I-\tilde{\Omega }\right)}^{-1}$,$e_{i}^{{}^{\prime }}=\left(0,\cdots ,1,\cdots ,0\right)$.

Therefore, the changes of sector-level markup or monopoly degree will affect the sales share and profits of other industries through the knowledge network, and furthermore affect the wage rate and total profit share. The more monopolistic an industry is, the lower wages will be, and the sensitivity of wages to industry markups depends solely on ${\bar{\beta }}_{i}$. This is intuitive, because as the concentration of sector *i* increases, the price of goods in that sector will rises, which in turn pushes up the marginal cost of production in downstream industries and increase the price of goods in downstream industries. As a result, the goods of sector *i* consumed by consumers directly or indirectly (through the network) determine the elasticity of real wages to the supplier centrality of sector *i*.

With the above two formulas, combined with the expression of actual output, we can find the elasticity of the total consumption level to the sector-level markup:

$$\begin{array}{c} \frac{\partial logC}{\partial log\mu_{i}}=-\frac{w}{w-\sum_{i=1}^{N}N_{i}\phi_{i}}\cdot {\bar{\beta }}_{i}+\frac{\mu_{i}}{1-{\tilde{\beta }}^{\prime }{\left\{1-\mu_{i}^{-1}\right\}}_{i}}\cdot \frac{\partial \left(\frac{Pro}{P_{C}C}\right)}{\partial \mu_{i}} \end{array}$$ (22)

The effect of industry concentration on total consumption is ambiguous, since an increase in industry concentration of sector *i* reduces real wages but also increases the share of profits. More strictly, as the concentration of sector *i* increases, there are two opposite effects: 1. Negative effects on wages (first term on the right side of the equation above); 2. Positive effect on profit share (second term on the right side of the equation). The intuition is as follows: 1. with the increase of the concentration of sector *i*, the price of consumption goods is affected directly and indirectly (through network transmission), thus leisure becomes relatively cheap, so consumers tend to reduce labor input and enjoy more leisure; 2. increased concentration pushes up aggregate profits, ultimately expanding consumers’ budget constraints and thus increasing consumption. So whether a rise in concentration improves or worsens consumer welfare lies between the trade-off of these two effects.

Take the expression of $\frac{\partial \left(\frac{Pro}{P_{C}C}\right)}{\partial \mu_{i}}$ into the equation above, with some algebraic transformations, we can obtain:

$$\begin{array}{c} \frac{\partial logC}{\partial log\mu_{i}}<{\tilde{\beta }}_{i}\left({\frac{\mu_{i}^{-1}}{1-{\tilde{\beta }}^{\prime }\left\{1-\mu_{i}^{-1}\right\}}}_{i}-1\right) \end{array}$$ (23)

When the degree of monopoly of industry *i*, *μ*_*i*_, is high enough, then ${\frac{\mu_{i}^{-1}}{1-{\tilde{\beta }}^{\prime }\left\{1-\mu_{i}^{-1}\right\}}}_{i}<1$, which leads to $\frac{\partial logC}{\partial log\mu_{i}}<0$. In other words, increasing (reducing) concentration levels in highly monopolistic industries does harm (enhance) consumers’ welfare. In the following, I will take vertical network, star network and non-existent knowledge network as examples to analyze the impact of changes in industry monopoly degree on the total welfare of consumers.

Take the vertical economy as an example: $w=\prod_{i}\mu_{i}^{-1}\cdot \varphi_{i}$ and $C=\frac{\prod_{i}\mu_{i}^{-1}\cdot \varphi_{i}-\sum_{i}N_{i}\cdot \phi_{i}}{\prod_{i}\mu_{i}^{-1}}=\prod_{i}\varphi_{i}-\prod_{i}\mu_{i}\cdot \sum_{i}N_{i}\cdot \phi_{i}$. We can see that the rise of market concentration reduces real output in vertical economies. That is to say, with the increase of industry monopoly, while the increase of profit increases the budget of consumers, the decline of wage level damages the welfare of consumers. The increased profit share cannot fully cover and make up for the loss, and finally the total welfare level of consumers decrease.

Take the star economy as an example:

$$w=\left(\mu_{1}^{-1}\varphi_{1}\right)\cdot {\left(\mu_{2}^{-1}\varphi_{2}\right)}^{\beta_{2}}\cdots {\left(\mu_{N}^{-1}\varphi_{N}\right)}^{\beta_{N}}$$

$$C=\frac{\left(\mu_{1}^{-1}\varphi_{1}\right)\cdot {\left(\mu_{2}^{-1}\varphi_{2}\right)}^{\beta_{2}}\cdots {\left(\mu_{N}^{-1}\varphi_{N}\right)}^{\beta_{N}}-\sum_{i}N_{i}\phi_{i}}{\left(\beta_{1}+\beta_{2}\mu_{2}^{-1}+\cdots +\beta_{N}\mu_{N}^{-1}\right)\cdot \mu_{1}^{-1}}$$

Take the without-network economy as an example:

$$w={\left(\mu_{1}^{-1}\varphi_{1}\right)}^{\beta_{1}}\cdot {\left(\mu_{2}^{-1}\varphi_{2}\right)}^{\beta_{2}}\cdots {\left(\mu_{N}^{-1}\varphi_{N}\right)}^{\beta_{N}}$$

$$C=\frac{{\left(\mu_{1}^{-1}\varphi_{1}\right)}^{\beta_{1}}\cdot {\left(\mu_{2}^{-1}\varphi_{2}\right)}^{\beta_{2}}\cdots {\left(\mu_{N}^{-1}\varphi_{N}\right)}^{\beta_{N}}-\sum_{i}N_{i}\phi_{i}}{\left(\beta_{1}\mu_{1}^{-1}+\beta_{2}\mu_{2}^{-1}+\cdots +\beta_{N}\mu_{N}^{-1}\right)}$$

An increase in fixed costs or an increase in market concentration will reduce the actual output of the economy. However, rising fixed costs will cause low-productivity firms to exit the industry, which will also increase the productivity of the industry, and therefore increase the actual output of the economy. Therefore, increasing fixed costs will improve or worsen consumer welfare depends on the impact of these two aspects.

In the following proposition, I establish a connection between the product market and the patent market.

**Proposition 6**

$$\begin{array}{c} \tilde{sale}\left(i,k\right)=s\left(i,k\right) \end{array}$$ (24)

Where $\tilde{sale}\left(i,k\right)$ is sales share of firm *k* in sector *i* and *s*(*i*, *k*) is firm *k*’s share of patents in sector *i*. The first thing to note in the above proposition is that: for a given firm in a specific sector, the market share of sales and patents are equal. As an inference, the industry concentration of product and patent markets are the same, consistent with **Fact 4**.

#### Idiosyncratic fixed cost

In the previous section, we have assumed that when a firm conduct research in a given sector, then this firm should pay a sector-specific fixed cost. However, for any given sector, the entry cost is not the same for all firms. For firms which have ever entered the sectors or have patents in close sectors, the entry cost is low compared with peripheral sectors. We further assume that firms pay a firm-specific fixed cost *φ*(*i*, *k*) instead of *φ*_*i*_. Now we can rewrite proposotion 3 as follows:

$$\begin{array}{c} \pi \left(i,k\right)=\frac{\mu \left(i,k\right)-1}{\mu \left(i,k\right)}s\left(i,k\right){\tilde{\beta }}_{i}P_{c}C-\phi \left(i,k\right) \end{array}$$ (25)

Firm *k* Would innovate in sector *i* only if *φ*(*i*, *k*) is low and sector-level supplier centrality is high enough. That is to say, firm *k* would innovate in sector *i* only if the sector firm *k* innovates in is close to sector *i*, and sector *i* is at the core of the knowledge network.

## Conclusion

This paper documents that over the last 20 years the level of R&D market concentration in the US has increased across most industries. I show that increasing barriers into patent markets have implications to firm performance, as they affect profitability, innovation, and returns to investors. First, the increase in entry costs is associated with small firms to exit. Since small scale firms have less profit to offset the enrty costs. Second, the remaining large firms generating higher profits and survival rates through higher market power. Consistent with the idea that market power considerations are becoming a key source of value during the increase in industry concentration levels. Finally, the increase in concentration levels consolidate the market power of the incumbent firms furthermore. So that they can enter into more sectors. Firms enter into new sectors by tradeoff market competition and entry cost. After entering the new sectors, these firms gradually consolidate their position in order to earn more profit and enter into more sectors. In general, our findings suggest that despite popular beliefs, competition could have been fading over time.

I bridge the gap between the product market and the R&D market. As interpreted in the model, on the one hand, large firms in the R&D market are still large firms in the product market, and large firms in the product market would charge higher price on their product so that they can charge higher profit which is a part of the value of their knowledge on the other hand.

More broadly, the findings that firms in industries that have become more concentrated generate higher profit margins, and enjoy better investment opportunities should be of interest to policy makers. While at least parts of these gains appear to be transferred to the firms’ shareholders, it is not clear whether the higher market concentration benefits consumers or other stakeholders. The increase in profit margins without a corresponding economically significant increase in efficiency may suggest the opposite. Although it is possible that a more concentrated nature of R&D and product markets improve the quality or variety of patents and products offered, it is unclear whether those changes are sufficient to compensate customers for the higher profit margins that firms enjoy. Our findings may motivate policy makers to examine the impact of the increased concentration further. This paper is a starting point for studying the quantitative implications of increasing concentration levels. What are the appropriate goverment policies to mitigate the potential inefficiencies? I leave these questions for future research.

## Appendix: Proofs

Proof of proposition 1: We define the sectoral theoretical price index and quantity index as:

$$\begin{array}{c} P_{i}={\left\{\sum_{k=1}^{M^{i}}P{\left(i,k\right)}^{1-\varepsilon_{i}}\right\}}^{\frac{1}{1-\varepsilon_{i}}},Z_{i}={\left\{\sum_{k=1}^{M^{i}}Z{\left(i,k\right)}^{\frac{\varepsilon_{i}-1}{\varepsilon_{i}}}\right\}}^{\frac{\varepsilon_{i}}{\varepsilon_{i}-1}} \end{array}$$ (26)

Solve the optimization problem:$P_{i}Z_{i}-\sum_{k=1}^{M^{i}}P\left(i,k\right)Z\left(i,k\right)$ we get:

$$\begin{array}{c} \frac{P\left(i,k\right)}{P_{i}}={\left[\frac{Z\left(i,k\right)}{Z_{i}}\right]}^{-\frac{1}{\varepsilon_{i}}} \end{array}$$ (27)

Define: $Z=\prod_{i=1}^{N}{\left(Z_{i}\right)}^{\beta_{i}}$, and $P=\prod_{i=1}^{K}{\left(P_{i}\right)}^{\beta_{i}}$.

Similarly, the first order condition satisfies:

$$\begin{array}{c} \frac{P_{i}}{P}=\beta_{i}{\left[\frac{Z_{i}}{Z}\right]}^{-1} \end{array}$$ (28)

Solve the profit maximization problem:

$$\begin{array}{c} max:\;P\left(i,k\right)Z\left(i,k\right)-\lambda \left(i,k\right)Z\left(i,k\right) \end{array}$$ (29)

subject to the above two demand functions.

From the above two demand functions, we can obtain:

$$\begin{array}{c} P\left(i,k\right)=Z{\left(i,k\right)}^{-\frac{1}{\varepsilon_{i}}}{\left(Z_{i}\right)}^{\frac{1}{\varepsilon_{i}}-1}\beta_{i}PZ \end{array}$$ (30)

Take derivative w.r.t. *Z*(*i*, *k*) and define the sales share:

$$\begin{array}{c} s\left(i,k\right)=\frac{P\left(i,k\right)Z\left(i,k\right)}{P_{i}Z_{i}} \end{array}$$ (31)

We can obtain the pricing rule in the proposition 1. So we complete the proof. Note that knowledge within a sector is more substitutable than knowledge across sectors: *ε*_*i*_ > 1. And firms play a static game of quantity competition. Specifically, each firm does recognize that sectoral prices and quantities vary when that firm changes its quantity.

Proof of corollary 1: According to the marginal cost function, when *φ*(*i*, *k*) = *φ*(*i*, *l*), we have λ(*i*, *k*) = λ(*i*, *l*). We suppose *s*(*i*, *k*) > *s*(*i*, *l*), then from the firm’s knowledge pricing functions we have *μ*(*i*, *k*) > *μ*(*i*, *l*) and *P*(*i*, *k*) > *P*(*i*, *l*). Since $s\left(i,k\right)={\left(\frac{P\left(i,k\right)}{P_{i}}\right)}^{1-\varepsilon_{i}}$, then *s*(*i*, *k*) < *s*(*i*, *l*), contradiction. If we suppose *s*(*i*, *k*) < *s*(*i*, *l*), similar to the logic above, we have contradiction. Then *φ*(*i*, *k*) = *φ*(*i*, *l*) implies *s*(*i*, *k*) = *s*(*i*, *l*). Similarly, *φ*(*i*, *k*) > *φ*(*i*, *l*) implies *s*(*i*, *k*) > *s*(*i*, *l*), and *φ*(*i*, *k*) < *φ*(*i*, *l*) implies *s*(*i*, *k*) < *s*(*i*, *l*).

Proof of lemma 1: To prove this lemma, I reproduce the Eq (11) here for convenience:

$$\begin{array}{c} \mu_{i}=\frac{P_{i}}{\lambda_{i}}={\left(\sum_{k=1}^{M^{i}}\mu {\left(i,k\right)}^{-1}s\left(i,k\right)\right)}^{-1} \end{array}$$ (32)

The markup charged by firm *i* in sector *k* is equal to $\mu_{t}\left(i,k\right)=\frac{\varepsilon_{i}}{\left(\varepsilon_{i}-1\right)\left[1-s\left(i,k\right)\right]}$. Let us substitute it into sectoral markup function above. After some simplification, we have:

$$\begin{array}{c} \begin{array}{cc} \mu_{i} & ={\left(\sum_{k=1}^{M^{i}}\frac{\left(\varepsilon_{i}-1\right)\left(1-s\left(i,k\right)\right)}{\varepsilon_{i}}s\left(i,k\right)\right)}^{-1}\\ & =\frac{\varepsilon_{i}}{\varepsilon_{i}-1}{\left\{\sum_{k=1}^{M^{i}}\left[1-s\left(i,k\right)\right]s\left(i,k\right)\right\}}^{-1}\\ & =\frac{\varepsilon_{i}}{\varepsilon_{i}-1}{\left\{1-\sum_{k=1}^{M^{i}}s{\left(i,k\right)}^{2}\right\}}^{-1},\sum_{k=1}^{M^{i}}s\left(i,k\right)=1\\ & =\frac{\varepsilon_{i}}{\varepsilon_{i}-1}{\left\{1-HHI_{i}\right\}}^{-1},HHI_{i}=\sum_{k=1}^{M^{i}}s_{i}{\left(i,k\right)}^{2} \end{array} \end{array}$$ (33)

Proof of proposition 2: The sector-level marginal cost is equal to

$$\begin{array}{c} \lambda_{i}=\varphi_{i}^{-1}w^{r_{i}}\prod_{j=1}^{N}P_{j}^{\omega_{ij}} \end{array}$$ (34)

Substitute

$$\begin{array}{c} \lambda_{i}=\frac{P_{i}}{\mu_{i}} \end{array}$$ (35)

We have

$$\begin{array}{c} P_{i}=\mu_{i}\varphi_{i}^{-1}w^{r_{i}}\prod_{j=1}^{N}P_{j}^{\omega_{ij}} \end{array}$$ (36)

Take logarithm of both sides of the equation to get

$$\begin{array}{c} logP_{i}=r_{i}\cdot logw+\sum_{j=1}^{N}\omega_{ij}logP_{j}+log\left(\mu_{i}\varphi_{i}^{-1}\right) \end{array}$$ (37)

Rewrite this in matrix form to get

$$\begin{array}{c} \left(I-\Omega \right){\left\{logP_{i}\right\}}_{i}={\left\{log\mu_{i}\varphi_{i}^{-1}w^{r_{i}}\right\}}_{i} \end{array}$$ (38)

Finally, we assume matrix (*I* − Ω) is invertible. Then it follows that

$$\begin{array}{c} {\left\{logP_{i}\right\}}_{i}={\left(I-\Omega \right)}^{-1}{\left\{log\mu_{i}\varphi_{i}^{-1}w^{r_{i}}\right\}}_{i} \end{array}$$ (39)

The market clearing condition for the variety *k* of sector *i*’s patents is such that the supply is equal to the demand from the household and from other firms in the economy:

$$\begin{array}{c} P\left(i,k\right)Z\left(i,k\right)=P\left(i,k\right)C\left(i,k\right)+\sum_{j}\sum_{l}P\left(i,k\right)Z\left(j,l,i,k\right) \end{array}$$ (40)

The household’s problem gives

$$\begin{array}{c} P\left(i,k\right)C\left(i,k\right)=\beta_{i}{\left(\frac{P\left(i,k\right)}{P_{i}}\right)}^{1-\varepsilon_{i}}P_{c}C \end{array}$$ (41)

The cost minimization problem gives

$$\begin{array}{c} P\left(i,k\right)Z\left(j,l,i,k\right)=\omega_{ji}{\left(\frac{P\left(i,k\right)}{P_{i}}\right)}^{1-\varepsilon_{i}}\lambda \left(j,l\right)Z\left(j,l\right) \end{array}$$ (42)

Summing over the firms in sector *i*, we have

$$\begin{array}{c} \begin{array}{cc} P_{i}Z_{i}=\sum_{k=1}^{M^{i}}P\left(i,k\right)Z\left(i,k\right) & =\beta_{i}P_{c}C+\sum_{j=1}^{N}\omega_{ji}\sum_{l=1}^{M^{l}}\lambda \left(j,l\right)Z\left(j,l\right)\\ & =\beta_{i}P_{c}C+\sum_{j=1}^{N}\omega_{ji}\mu_{j}^{-1}P_{j}Z_{j} \end{array} \end{array}$$ (43)

where in the last equality, I use the definition of the sector marginal cost λ_*j*_ and the fact that $\lambda_{j}=\mu_{j}^{-1}P_{j}$. The above equation in matrix form thus yields

$$\begin{array}{c} {\left\{\frac{P_{i}Z_{i}}{P_{c}C}\right\}}_{i}^{\prime }=\beta^{\prime }{\left(I-\tilde{\Omega }\right)}^{-1}={\tilde{\beta }}^{\prime } \end{array}$$ (44)

Proof of proposition 3: Note that the profits of firm *k* in sector *i* are

$$\begin{array}{c} \pi \left(i,k\right)=P\left(i,k\right)Z\left(i,k\right)-\lambda \left(i,k\right)Z\left(i,k\right)-\phi_{i} \end{array}$$ (45)

This is equivalent to

$$\begin{array}{c} \begin{array}{cc} \pi \left(i,k\right) & =P\left(i,k\right)Z\left(i,k\right)-\frac{P\left(i,k\right)}{\mu \left(i,k\right)}Z\left(i,k\right)-\phi_{i}\\ & =\frac{\mu \left(i,k\right)-1}{\mu \left(i,k\right)}P\left(i,k\right)Z\left(i,k\right)-\phi_{i} \end{array} \end{array}$$ (46)

By definition of patent share and supplier centrality

$$\begin{array}{c} s\left(i,k\right)=\frac{P\left(i,k\right)Z\left(i,k\right)}{P_{i}Z_{i}}\;,\;{\left\{\frac{P_{i}Z_{i}}{P_{c}C}\right\}}_{i}^{\prime }=\beta^{\prime }{\left(I-\tilde{\Omega }\right)}^{-1}={\tilde{\beta }}^{\prime } \end{array}$$ (47)

Rearrange to get the desired result:

$$\begin{array}{c} \pi \left(i,k\right)=\frac{\mu \left(i,k\right)-1}{\mu \left(i,k\right)}s\left(i,k\right){\tilde{\beta }}_{i}P_{c}C-\phi_{i} \end{array}$$ (48)

Proof of proposition 4: We first consider the case that all existing firms’ market share expand the same scale multiplier *α* > 1: ${\tilde{s}}_{i}=s_{i}\cdot \alpha$, for all 1 ≤ *i* ≤ *k*. Then

$$\begin{array}{c} \sum_{i=1}^{k}s_{i}=\sum_{i=1}^{k}\frac{{\tilde{s}}_{i}}{\alpha }=\frac{1}{\alpha } \end{array}$$ (49)

Then, it follows that

$$\begin{array}{c} \mu_{k}=\sum_{i=1}^{k}{\left(s_{i}\cdot \alpha \right)}^{2}=\alpha^{2}\cdot \sum_{i=1}^{k}s_{i}^{2}=\frac{\sum_{i=1}^{k}s_{i}^{2}}{{\left(\sum_{i=1}^{k}s_{i}\right)}^{2}} \end{array}$$ (50)

Since, according to defintion, $\sum_{i=1}^{n}s_{i}=1$

$$\begin{array}{c} \mu_{n}=\sum_{i=1}^{n}s_{i}^{2}=\frac{\sum_{i=1}^{n}s_{i}^{2}}{{\left(\sum_{i=1}^{n}s_{i}\right)}^{2}} \end{array}$$ (51)

This means that we just need to prove

$$\begin{array}{c} \frac{\sum_{i=1}^{k}s_{i}^{2}}{{\left(\sum_{i=1}^{k}s_{i}\right)}^{2}}>\frac{\sum_{i=1}^{n}s_{i}^{2}}{{\left(\sum_{i=1}^{n}s_{i}\right)}^{2}} \end{array}$$ (52)

We prove this inequality by two steps. In the first step, we prove

$$\begin{array}{c} \frac{\sum_{i=1}^{n-1}s_{i}^{2}}{{\left(\sum_{i=1}^{n-1}s_{i}\right)}^{2}}>\frac{\sum_{i=1}^{n}s_{i}^{2}}{{\left(\sum_{i=1}^{n}s_{i}\right)}^{2}} \end{array}$$ (53)

In the second step, we prove

$$\begin{array}{c} \frac{\sum_{i=1}^{k-1}s_{i}^{2}}{{\left(\sum_{i=1}^{k-1}s_{i}\right)}^{2}}>\frac{\sum_{i=1}^{k}s_{i}^{2}}{{\left(\sum_{i=1}^{k}s_{i}\right)}^{2}},\;for\;all\;1<k\leq n \end{array}$$ (54)

(i): To prove the first inequality, we just need to prove

$$\begin{array}{c} \frac{\sum_{i=1}^{n-1}s_{i}^{2}}{{\left(1-s_{n}\right)}^{2}}>\frac{\sum_{i=1}^{n}s_{i}^{2}}{1} \end{array}$$ (55)

Rearrange this to get

$$\begin{array}{c} \sum_{i=1}^{n-1}s_{i}^{2}>\left(\sum_{i=1}^{n-1}s_{i}^{2}+s_{n}^{2}\right)\left(1-2s_{n}+s_{n}^{2}\right) \end{array}$$ (56)

After simplification, we just need to prove

$$\begin{array}{c} 2\sum_{i=1}^{n}s_{i}^{2}>s_{n}\left(\sum_{i=1}^{n}s_{i}^{2}+1\right) \end{array}$$ (57)

That is

$$\begin{array}{c} \sum_{i=1}^{n}s_{i}^{2}+\sum_{i=1}^{n}s_{i}^{2}>s_{n}\sum_{i=1}^{n}s_{i}^{2}+s_{n} \end{array}$$ (58)

Finally, note that

$$\begin{array}{c} \sum_{i=1}^{n}s_{i}^{2}>\frac{1}{n}>s_{n} \end{array}$$ (59)

Then we complete the first step.

(ii): To prove the second inequality, we just need to prove

$$\begin{array}{c} \frac{\sum_{i=1}^{k-1}{\left(\alpha \cdot s_{i}\right)}^{2}}{{\left(\sum_{i=1}^{k-1}\alpha \cdot s_{i}\right)}^{2}}>\frac{\sum_{i=1}^{k}{\left(\alpha \cdot s_{i}\right)}^{2}}{1} \end{array}$$ (60)

Note that $\sum_{i=1}^{k-1}\alpha \cdot s_{i}=1$, and conbine with the result in the first step. The we complete the second step.

Finally, we relax the constant scale multiplier assumption. We consider two firms with market share *s*_1_ > *s*_2_. New market shares are *α*_1_ *s*_1_ and *α*_2_ *s*_2_. Suppose 1 < *α*_1_ ≤ *α*_2_. Then

$$\begin{array}{c} \frac{s_{1}}{s_{2}}\geq \frac{\alpha_{1}s_{1}}{\alpha_{2}s_{2}} \end{array}$$ (61)

By definition

$$\begin{array}{c} s_{1}=\frac{P_{1}Z_{1}}{PZ}\;and\;s_{2}=\frac{P_{2}Z_{2}}{PZ} \end{array}$$ (62)

$$\begin{array}{c} \alpha_{1}s_{1}=\frac{{\tilde{P}}_{1}Z_{1}}{\tilde{P}\tilde{Z}}\;and\;\alpha_{2}s_{2}=\frac{{\tilde{P}}_{2}Z_{2}}{\tilde{P}\tilde{Z}} \end{array}$$ (63)

Rearrange and simplify to get

$$\begin{array}{c} \frac{P_{1}}{P_{2}}\geq \frac{{\tilde{P}}_{1}}{{\tilde{P}}_{2}} \end{array}$$ (64)

According to firm’s pricing rule, this implies that

$$\begin{array}{c} \frac{P_{1}}{P_{2}}=\frac{1-s_{2}}{1-s_{1}}\;and\;\frac{{\tilde{P}}_{1}}{{\tilde{P}}_{2}}=\frac{1-\alpha_{2}s_{2}}{1-\alpha_{1}s_{1}} \end{array}$$ (65)

Then we get

$$\begin{array}{c} \frac{1-s_{2}}{1-\alpha_{2}s_{2}}\geq \frac{1-s_{1}}{1-\alpha_{1}s_{1}} \end{array}$$ (66)

It’s contradictory with *s*_1_ > *s*_2_. Then we have *α*_1_ > *α*_2_, which means that larger firms expand more than smaller firms. As a result, sector-level concentration increases more than constant scale multiplier case.

Proof of proposition 5: Without loss of generality, let us normalize the price of conposite consumption good to *P*_*c*_ = 1. It implies

$$\begin{array}{c} \begin{array}{cc} 0 & =\beta^{\prime }{\left(I-\Omega \right)}^{-1}{\left\{log\mu_{i}\varphi_{i}^{-1}w^{r_{i}}\right\}}_{i}\\ & =\beta^{\prime }{\left(I-\Omega \right)}^{-1}{\left\{r_{i}\right\}}_{i}logw+\beta^{\prime }{\left(I-\Omega \right)}^{-1}{\left\{log\mu_{i}\varphi_{i}^{-1}\right\}}_{i} \end{array} \end{array}$$ (67)

Note that

$$\begin{array}{c} \Omega I={\left\{\sum_{l=1}^{N}\omega_{kl}\right\}}_{k}={\left\{1-r_{k}\right\}}_{k}=I-{\left\{r_{k}\right\}}_{k} \end{array}$$ (68)

where $I={\left\{1\right\}}_{k}$ is the vector of ones. It implies that ${\left(I-\Omega \right)}^{-1}{\left\{r_{l}\right\}}_{l}=I$. Furthermore, because $\sum_{i=1}^{N}\beta_{i}=1$, it follows that

$$\begin{array}{c} \beta^{\prime }{\left(I-\Omega \right)}^{-1}{\left\{r_{l}\right\}}_{l}=\beta^{\prime }I=\sum_{i=1}^{N}\beta_{i}=1 \end{array}$$ (69)

Thus we have the expression of the wage

$$\begin{array}{c} log\;w=-\beta^{\prime }{\left(I-\Omega \right)}^{-1}{\left\{log\;\mu_{i}\varphi_{i}^{-1}\right\}}_{i} \end{array}$$ (70)

From the firm’s problem, it is clear that the profit *π*(*i*, *k*) of firm *k* in sector *i* is such that *π*(*i*, *k*) = *P*(*i*, *k*)*Z*(*i*, *k*) − λ(*i*, *k*)*Z*(*i*, *k*). Summing over the firms in sector *i* yields:

$$\begin{array}{c} \pi_{i}=\sum_{k=1}^{N^{i}}P\left(i,k\right)Z\left(i,k\right)-\sum_{k=1}^{N^{i}}\lambda \left(i,k\right)Z\left(i,k\right)=P_{i}Z_{i}-\lambda_{i}Z_{i}=\left(1-\mu_{i}^{-1}\right)P_{i}Z_{i} \end{array}$$ (71)

where I use the definition of the marginal cost in sector *i* and the fact that $\lambda_{i}=\mu_{i}^{-1}P_{i}$. Finally, aggregate profit is equal to the sum of the profit in each sector:

$$\begin{array}{c} \frac{Pro}{P_{c}C}=\sum_{i=1}^{N}\frac{\pi_{i}}{P_{c}C}=\sum_{i=1}^{N}\left(1-\mu_{i}^{-1}\right)\frac{P_{i}Z_{i}}{P_{c}C}={\left\{\frac{P_{i}Z_{i}}{P_{c}C}\right\}}_{i}^{\prime }{\left\{1-\mu_{i}^{-1}\right\}}_{i} \end{array}$$ (72)

Substitute

$$\begin{array}{c} {\left\{\frac{P_{i}Z_{i}}{P_{c}C}\right\}}_{i}^{\prime }=\beta^{\prime }{\left(I-\tilde{\Omega }\right)}^{-1}={\tilde{\beta }}^{\prime } \end{array}$$ (73)

Then we get the desired result.

The household budget constraint is such that total expenditure is equal to the labor and profit income:

$$\begin{array}{c} P_{c}C=wL+Pro\Leftrightarrow C=w+\frac{Pro}{P_{c}C}C \end{array}$$ (74)

Rearrange terms and take logs:

$$\begin{array}{c} log\;C=log\;w-log\left(1-\frac{Pro}{P_{c}C}\right) \end{array}$$ (75)

where I use the normalization of the price *P*_*c*_ = 1 and the labor *L* = 1.

$$\begin{array}{c} logC=log\left(w-\sum_{i=1}^{N}N^{i}\phi_{i}\right)-log\left(1-\tilde{\beta }{\left\{1-\mu_{i}^{-1}\right\}}_{i}\right) \end{array}$$ (76)

$$\begin{array}{c} logw=-\tilde{\beta }\left\{log\mu_{i}\varphi_{i}^{-1}\right\} \end{array}$$ (77)

Proof of proposition 6:

From proposition 2, we can obtain:

$$\begin{array}{c} P\left(i,k\right)C\left(i,k\right)=\beta_{i}{\left(\frac{P\left(i,k\right)}{P_{i}}\right)}^{1-\varepsilon_{i}}P_{c}C \end{array}$$ (78)

$$\begin{array}{c} P\left(i,k\right)Z\left(j,l,i,k\right)=\omega_{ji}{\left(\frac{P\left(i,k\right)}{P_{i}}\right)}^{1-\varepsilon_{i}}\lambda \left(j,l\right)Z\left(j,l\right) \end{array}$$ (79)

$$\begin{array}{c} \sum_{j}\sum_{l}P\left(i,k\right)Z\left(j,l,i,k\right)=\sum_{j}\omega_{ji}{\left(\frac{P\left(i,k\right)}{P_{i}}\right)}^{1-\varepsilon_{i}}\mu_{j}^{-1}P_{j}Z_{j} \end{array}$$ (80)

Then

$$\begin{array}{c} \frac{C\left(i,k\right)}{Z\left(i,k\right)}=\frac{\beta_{i}}{{\tilde{\beta }}_{i}} \end{array}$$ (81)

$$\begin{array}{c} \tilde{sale}\left(i,k\right)=\frac{P\left(i,k\right)C\left(i,k\right)}{\sum_{k}P\left(i,k\right)C\left(i,k\right)}=\frac{P\left(i,k\right)Z\left(i,k\right)}{\sum_{k}P\left(i,k\right)Z\left(i,k\right)}=s\left(i,k\right) \end{array}$$ (82)

## Supporting information

S1 Data R&D data record the concentration of different industries in different years. The raw data is from NBER patent project.
(DTA)

## References

[1] Gustavo Grullon, Yelena Larkin, Roni Michaely. Are US Industries Becoming More Concentrated? Review of Finance, Volume 23, Issue 4, July 2019, Pages 697–743.

[2] Hsieh CT, Rossi-Hansberg E. The industrial revolution in services. National Bureau of Economic Research, 2019. Available from: 10.3386/w25968.

[3] Hopenhayn H, Neira J, Singhania R. From population growth to firm demographics: Implications for concentration, entrepreneurship and the labor share. National Bureau of Economic Research, 2018. Available from: 10.3386/w25382.

[4] Cohen WM, Klepper S. The anatomy of industry R&D intensity distributions. The American Economic Review, 1992: 773–799.

[5] Cohen Wesley M., and Klepper Steven. A Reprise of Size and R&D. The Economic Journal 106, no.437 (1996):925–51. doi: 10.2307/2235365

[6] Gu L. Product market competition, R&D investment, and stock returns. Journal of Financial Economics, 2016, 119(2): 441–455. doi: 10.1016/j.jfineco.2015.09.008

[7] Park Jungsoo. International and intersectoral R&D spillovers in the OECD and East Asian economies. Economic Inquiry 42.4 (2004): 739–757. doi: 10.1093/ei/cbh093

[8] Dixit AK, Stiglitz JE. Monopolistic competition and optimum product diversity. The American economic review, 1977, 67(3): 297–308.

[9] Atkeson A, Burstein A. Pricing-to-market, trade costs, and international relative prices. American Economic Review, 2008, 98(5): 1998–2031. doi: 10.1257/aer.98.5.1998

[10] Autor D., Dorn D., Katz L. F., Patterson C., & Van Reenen J. The fall of the labor share and the rise of superstar firms. The Quarterly Journal of Economics, 2020, 135(2), 645–709. doi: 10.1093/qje/qjaa004

[11] Gutiérrez G, Philippon T. The failure of free entry. National Bureau of Economic Research, 2019. Available from: 10.3386/w26001.

[12] Crouzet N, Eberly JC. Understanding weak capital investment: The role of market concentration and intangibles. National Bureau of Economic Research, 2019. Available from: 10.3386/w25869.

[13] Behrens K, Murata Y. General equilibrium models of monopolistic competition: a new approach. Journal of Economic Theory, 2007, 136(1): 776–787. doi: 10.1016/j.jet.2006.10.001

[14] Zhelobodko E., Kokovin S., Parenti M., & Thisse J. F. Monopolistic competition: Beyond the constant elasticity of substitution. Econometrica, 2012, 80(6): 2765–2784. doi: 10.3982/ECTA9986

[15] Melitz MJ, Ottaviano GIP. Market size, trade, and productivity. The Review of Economic Studies, 2008, 75(1): 295–316. doi: 10.1111/j.1467-937X.2007.00463.x

[16] Demidova S. Trade policies, firm heterogeneity, and variable markups. Journal of International Economics, 2017, 108: 260–273. doi: 10.1016/j.jinteco.2017.05.011

[17] Boucekkine R, Latzer H, Parenti M. Variable markups in the long-run: a generalization of preferences in growth models. Journal of Mathematical Economics, 2017, 68: 80–86. doi: 10.1016/j.jmateco.2016.11.005

[18] Grassi B. Io in io: Size, industrial organization, and the input-output network make a firm structurally important. Work. Pap., Bocconi Univ., Milan, Italy, 2017. Available from: https://repec.unibocconi.it/igier/igi/igier@unibocconi.it.

[19] Bernard AB, Redding SJ, Schott PK. Multiple-product firms and product switching. American Economic Review, 2010, 100(1): 70–97. doi: 10.1257/aer.100.1.70

[20] Eckel C, Neary JP. Multi-product firms and flexible manufacturing in the global economy. The Review of Economic Studies, 2010, 77(1): 188–217. doi: 10.1111/j.1467-937X.2009.00573.x

[21] Klette TJ, Kortum S. Innovating firms and aggregate innovation. Journal of Political Economy, 2004, 112(5): 986–1018. doi: 10.1086/422563

[22] Akcigit U, Kerr WR. Growth through heterogeneous innovations. Journal of Political Economy, 2018, 126(4): 1374–1443. doi: 10.1086/697901

[23] Irvine PJ, Pontiff J. Idiosyncratic return volatility, cash flows, and product market competition. The Review of Financial Studies, 2009, 22(3): 1149–1177. doi: 10.1093/rfs/hhn039

[24] Hall BH, Jaffe AB, Trajtenberg M. The NBER patent citation data file: Lessons, insights and methodological tools[J]. 2001. The NBER patent citation data file: Lessons, insights and methodological tools. National Bureau of Economic Research, 2001. Available from: 10.3386/w8498.

[25] Gutiérrez G, Jones C, Philippon T. Entry costs and the macroeconomy. National Bureau of Economic Research, 2019. Available from: 10.3386/w25609.

[26] Al‐Ubaydli O, McLaughlin PA. RegData: A numerical database on industry‐specific regulations for all United States industries and federal regulations, 1997–2012. Regulation & Governance, 2017, 11(1): 109–123. doi: 10.1111/rego.12107

[27] Cai J, Li N. Growth through inter-sectoral knowledge linkages. The Review of Economic Studies, 2019, 86(5): 1827–1866. doi: 10.1093/restud/rdy062

[28] Baqaee DR. Cascading failures in production networks. Econometrica, 2018, 86(5): 1819–1838. doi: 10.3982/ECTA15280

